# Chromosome-level genome assembly of the deep-sea snail *Phymorhynchus buccinoides* provides insights into the adaptation to the cold seep habitat

**DOI:** 10.1186/s12864-023-09760-0

**Published:** 2023-11-10

**Authors:** Zhaoqun Liu, Yuting Huang, Hao Chen, Chang Liu, Minxiao Wang, Chao Bian, Lingling Wang, Linsheng Song

**Affiliations:** 1 Liaoning Key Laboratory of Marine Animal Immunology, Dalian Ocean University, Dalian, 116023 China; 2 Functional Laboratory of Marine Fisheries Science and Food Production Processes, Qingdao National Laboratory for Marine Science and Technology, Qingdao, 266235 China; 3 Liaoning Key Laboratory of Marine Animal Immunology and Disease Control, Dalian Ocean University, Dalian, 116023 China; 4 Dalian Key Laboratory of Aquatic Animal Disease Prevention and Control, Dalian Ocean University, Dalian, 116023 China; 5 Laboratory of Aquatic Genomics, College of Life Sciences and Oceanography, Shenzhen University, Shenzhen, 518060 China; 6 College of Life Sciences, University of Chinese Academy of Sciences, Beijing, 100049 China; 7 Center of Deep Sea Research, and CAS Key Laboratory of Marine Ecology and Environmental Sciences, Institute of Oceanology, Chinese Academy of Sciences, Qingdao, 266071 China

**Keywords:** Cold seep, *Phymorhynchus buccinoides*, Chromosome-level genome, Glutamate regulation, Olfactory/chemosensory, H_2_S detoxification

## Abstract

**Background:**

The deep-sea snail *Phymorhynchus buccinoides* belongs to the genus *Phymorhynchus* (Neogastropoda: Raphitomidae), and it is a dominant specie in the cold seep habitat. As the environment of the cold seep is characterized by darkness, hypoxia and high concentrations of toxic substances such as hydrogen sulfide (H_2_S), exploration of the diverse fauna living around cold seeps will help to uncover the adaptive mechanisms to this unique habitat. In the present study, a chromosome-level genome of *P. buccinoides* was constructed and a series of genomic and transcriptomic analyses were conducted to explore its molecular adaptation mechanisms to the cold seep environments.

**Results:**

The assembled genome size of the *P. buccinoides* was approximately 2.1 Gb, which is larger than most of the reported snail genomes, possibly due to the high proportion of repetitive elements. About 92.0% of the assembled base pairs of contigs were anchored to 34 pseudo‐chromosomes with a scaffold N50 size of 60.0 Mb. Compared with relative specie in the shallow water, the glutamate regulative and related genes were expanded in *P. buccinoides*, which contributes to the acclimation to hypoxia and coldness. Besides, the relatively high mRNA expression levels of the olfactory/chemosensory genes in osphradium indicate that *P. buccinoides* might have evolved a highly developed and sensitive olfactory organ for its orientation and predation. Moreover, the genome and transcriptome analyses demonstrate that *P. buccinoides* has evolved a sulfite-tolerance mechanism by performing H_2_S detoxification. Many genes involved in H_2_S detoxification were highly expressed in ctenidium and hepatopancreas, suggesting that these tissues might be critical for H_2_S detoxification and sulfite tolerance.

**Conclusions:**

In summary, our report of this chromosome-level deep-sea snail genome provides a comprehensive genomic basis for the understanding of the adaptation strategy of *P. buccinoides* to the extreme environment at the deep-sea cold seeps.

**Supplementary Information:**

The online version contains supplementary material available at 10.1186/s12864-023-09760-0.

## Background

Deep-sea cold seeps are submarine springs where fluids emanate from the sea floor through the sediments by pressure gradients [1, 2]. The environment of deep-sea cold seeps is characterized by darkness, coldness, hypoxia, lack of photosynthesis-derived nutrients but rich in heavy metals and toxic substances [3–5]. There is no penetration of light in the 1000 m depth below the sea surface [6]. Low-oxygen zones also occur in the deeper waters of tropical and temperate oceans, usually between 100 and 1000 m [7]. Temperature in the oceans decreases with increasing depth. At tropical latitudes, the temperature range extends from 26 °C at the sea surface to 5 °C at 1500 m depth [8, 9]. In particular, the anaerobic oxidation of methane (AOM) via sulfate reduction is considered as the most important biogeochemical process at cold seeps [10]. During this process, carbonates are formed through anaerobic methane oxidation to produce extremely high concentrations of H_2_S in pore waters [11–13]. Therefore, an in-depth exploration of communities living in the deep-sea cold seeps will contribute to reveal the adaptation mechanism of this unique ecosystem.

In recent years, the benthic communities at the cold seeps have attracted increasing attention with the development of technologies for deep-sea research [14, 15]. A typical cold seep environment supports various communities of metazoans containing chemoautotrophic bacteria, tubeworms [16] and mussels [17]. As light disappears below 1000 m, deep-sea ​​organisms living in the dark communicate and interact through chemical signals [18]. The temperatures of cold seeps are 2 ℃ to 5 ℃ and the known adaptations of low temperature for other invertebrates are mainly about freezing below 0 ℃ [19, 20]. The cold seep habitat is characterized by chronic hypoxia, sometimes reaching complete anoxia. Organisms inhabiting these environments often adopt morphological adaptations of gills [21]. In particular, recent research has shown that the H_2_S level in the bottom water of the chemosynthetic communities is remarkably high (~ 1940 μM) at the active cold seep at Formosa Ridge (Site F) on the continental slope of the northern South China Sea [22]. The high concentrations of H_2_S will induce oxidative damage to the marine invertebrates such as molluscs [23]. Invertebrates achieve sulfide detoxification by oxidation of sulfide and thiosulfate is the main detoxification product [24–26]. Meanwhile, more and more high-throughput technologies and comparative genomics are shedding light on the researches of extreme environments adaptation [27, 28]. Since seep animals need to evolve unique adaptation mechanism to survive in the threatening environment, studying seep organisms at the genomic levels, especially the acclimation to the cold, dark, hypoxia, and H_2_S-rich environment, will help to discover novel physiological and biochemical capabilities, and provide clues to understand their adaptation and evolution strategies to thrive in the extreme environments [3, 29].

The snail *Phymorhynchus buccinoides*, which belongs to the order Neogastropoda was sampled from the active deep-sea cold seep of the South Sea of China in this research. Neogastropods are often dominant species of the benthic community at the top of the food chains due to their amazing predatory specializations [30]. Previous researches have also shown that as a secondary consumer, *P. buccinoides* feeds on mytilid mussel *Bathymodiolus platifrons*, biological carcass, and organic debris sinking down from the upper layer. It is the important predator of the food chains in the cold seeps and it contributes to the balances of the cold seep fauna communities, energy flows and other interactions among the communities living around the deep-sea cold seeps [31, 32]. In the present study, we report the first chromosome-level reference genome of the deep-sea snail *P. buccinoides*. Comparative genomic analyses of gene expansion, contraction, and identification of genes related to sulfate metabolism, chemical sensing, and glutamate regulative genes which contribute to the acclimation to hypoxia and coldness were also conducted, helping to elucidate the molecular basis of the adaptation to the deep-sea cold seep habitats.

## Results

### Sequencing, assembly, annotation of chromosome-scale genome

**Fig. 1:**
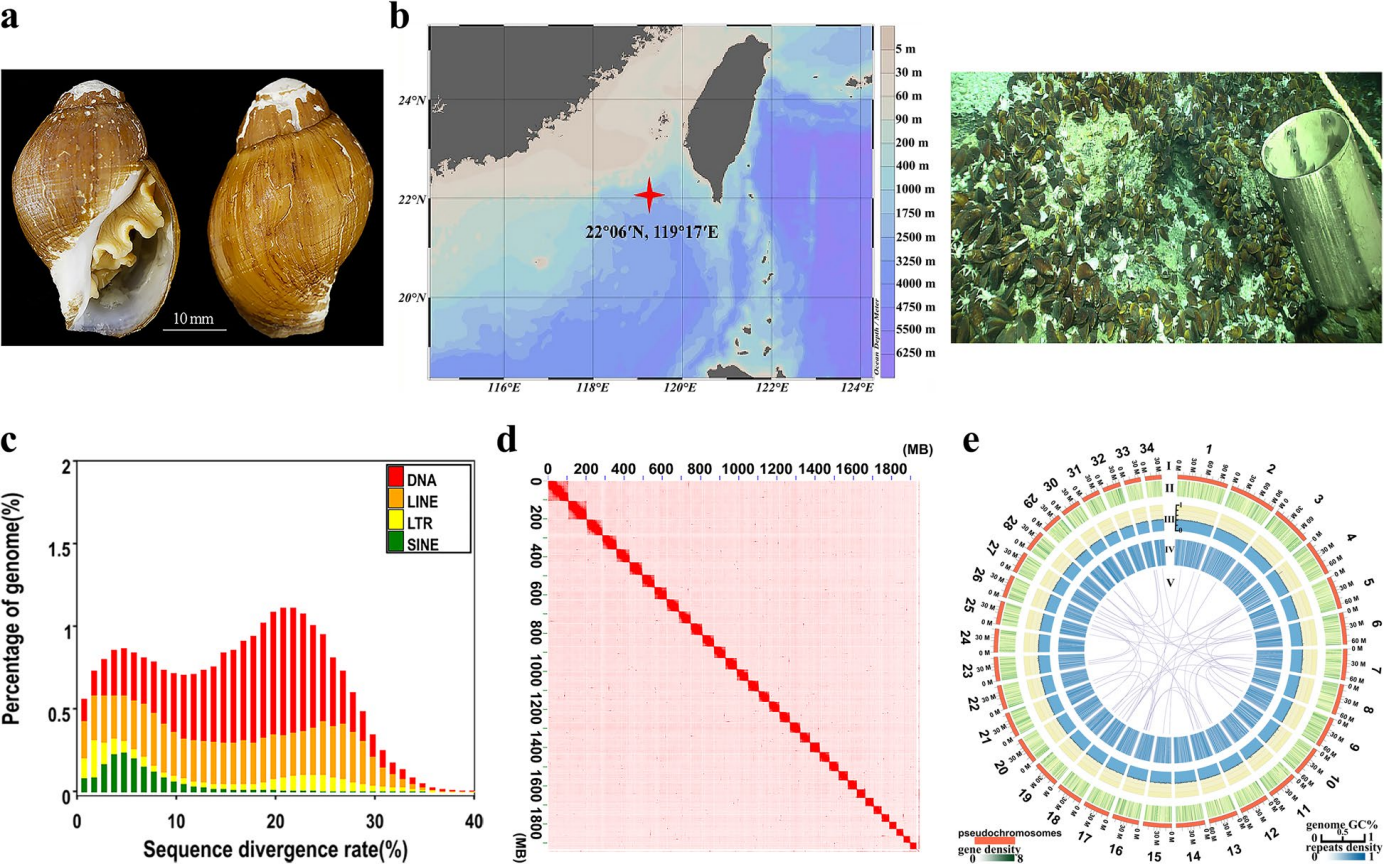
Sampling site, morphology, divergence distribution of transposable elements, chromosomal contact map and genomic landscape of *P. buccinoides*. **a** Morphology of *P. buccinoides*. **b** Sampling site of *P. buccinoides*. *P. buccinoides* used in the present study were collected from cold spring district. This specie is also common in cold spring vents. The mussels associated with *P. buccinoides* are also in photo. **c** Divergence distribution of transposable elements (TEs) in the *P. buccinoides* genome. De novo prediction. The distribution of sequence divergence rates of TEs as percentages of the genome size is shown. The y-axis shows the percentage of the genome that is annotated as TEs (TE contents). The x-axis shows sequence divergence rate. DNA transposon shown as DNA is indicated with red color, long interspersed nuclear element (LINE) is indicated with orange color, long terminal repeat (LTR) is indicated with yellow color, and short interspersed nuclear element (SINE) is indicated with green color. The percentage of genome and the sequence divergence rate show the cumulative proportion and inconsistency of repeat elements, respectively. **d** Chromosomal contact map of *P. buccinoides*. Based on Hi-C data, the chromosomal contact map was built. The contacts between one location and another are referred by blocks. The blocks correspond to 34 Chrs of *P. buccinoides*. The color reflects the intensity of each contact, and it represents the interaction density from high (red) to low (white) in the plot. In the x-axis and y-axis, each number means the genomic length (Mb). **e** Diagram and genomic landscape of the gastropod *P. buccinoides*. Circos atlas represents the Chr information of *P. buccinoides*. From outside to inside of the concentric circles, (I) Chr length (Mb) and numbers, (II) Density of gene distribution in each 100 kb genomic interval, (III) GC content of 100 kb genomic intervals, and (IV) distribution of genomic repeats density in 100 kb non-overlapping windows. Deep blue color indicates higher repeat density. (V) Major interchromosomal relationships of *P. buccinoides* Chrs are presented with purple lines, and each line indicates one pair of paralog genes

The genome of *P. buccinoides* (Fig. 1a) was sequenced using a hybrid approach. A total of raw reads and clean data including 52.2 Gb and 42.0 Gb for Illumina reads with an insert size of 350 bp (Table S1), 392.9 Gb and 327.1 Gb for Hi-C reads with insert sizes of 300–500 bp (Table S3), 47.5 Gb and 45.7 Gb for transcriptomic Illumina reads (Table S4), polymerase and subreads: 130.4 Gb and 130.1 Gb for genome (Table S2), 21.3 and 20.4 for transcriptome (Table S5) of Pacific Biosciences (PacBio) reads with a long insert size of 20 kb, were obtained using the NovaSeq 6000 platform and PacBio Sequel instrument (Table S7). The PLATANUS v1.2.4 and DBG2OLC were employed to assemble sequence reads into contig level [33, 34]. The total length of the assembled contigs of *P. buccinoides* was 2.1 Gb, and the contig N50 value was 308.7 kb (Table S8). The Benchmarking Universal Single-Copy Orthologs (BUSCO) value was 86.0% (total BUSCO groups for searching is 5295), indicating the completeness of the assembly. The technology of pseudo-chromosomes (Chrs) construction for assistant assembly was employed to produce the final chromosome-level genome. A total of 18,751 contigs were broken, clustered, ordered and mounted successfully in 34 Chrs (Fig. 1d, e). Finally, the chromosome-level assembly of *P. buccinoides* was obtained and the Hi-C contact map was also produced (Table S9 and Fig. 1d). The longest Chr was 105.8 Mb and the shortest was 34.0 Mb. The total length of the *P. buccinoides* final chromosomal-level assembled genome and Chrs sequences were 2.1 Gb and 1.9 Gb, respectively. About 92.0% of the assembled base pairs of contigs were anchored to Chrs, and the N50 size of Chrs was 60.0 Mb (Table S9). There were numerous links between Chrs of *P. buccinoides* and that of its relative species (Figure S2). Detailed distributions of gene density, GC content, repeat sequence content of each Chr, and the major inner connections in *P. buccinoides* Chrs were illustrated in Fig. 1e.

**Fig. 2:**
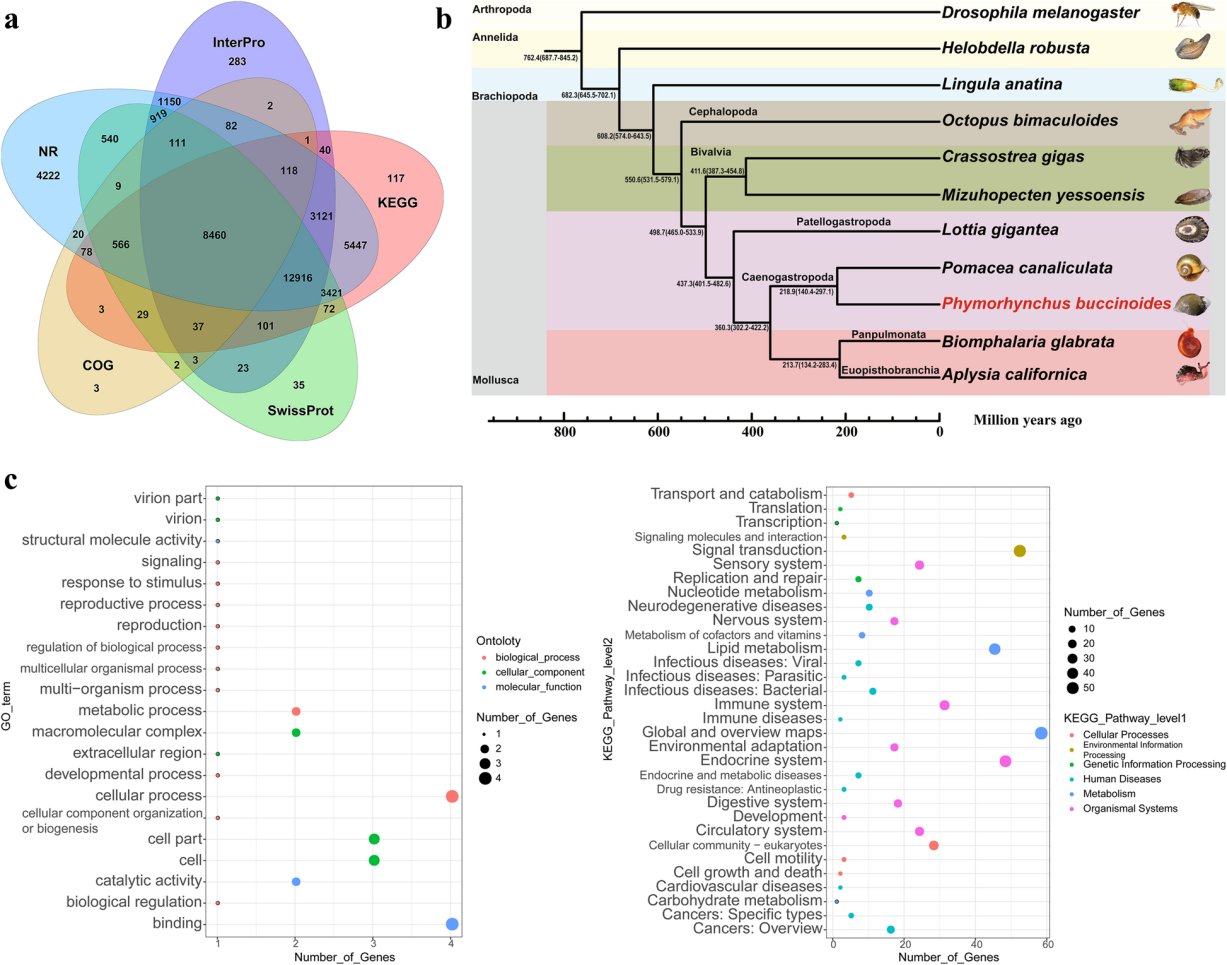
Venn, phylogeny and clusters of Gene Ontology (GO) and Kyoto Encyclopedia of Genes and Genomes (KEGG) enrichment analysis. **a** Venn of the annotation in *P. buccinoide*s. **b** Phylogenetic tree of *P. buccinoides*. The phylogenetic tree was based on the genome sequences. The reference divergence times for calibrations were retrieved from the TimeTree database. The black numbers on the branches represent the estimated diverge times. The *P. buccinoides* is marked in red color. **c** GO and KEGG enrichment analysis of gene families specific to *P. buccinoides*. The x-axis shows the number of genes and the y-axis shows the annotation terms. Different sizes and colors of bubbles exhibit different number and terms

In the assembled genome, the repeat content accounted for about 73.4% (Table S10). The transposable elements (TEs) including DNA transposons (24.19%) and retrotransposons (24.77%) accounted for 48.96% of the genome (Table S11), which showed high divergence (Fig. 1c, Figure S1). The retrotransposons were composed of 15.97% long interspersed nuclear elements (LINE), 1.74% short interspersed nuclear elements (SINE), and 7.06% long terminal repeats (LTR) (Table S11). A final nonredundant consensus gene set of *P. buccinoides* was obtained with the gene prediction and functional annotation (Fig. 2a). In the assembled genome, 45,545 protein-coding genes were predicted (Table S12), and 42,162 (92.6%) of them were functionally annotated (Table S13).

### Gene families and phylogeny of *P. buccinoides*

In the present study, the gene family cluster analysis of *P. buccinoides* and other 10 selected species (including 7 molluscan species) were performed. In total, 33,107 gene families and 100 single-copy orthologs were identified across *P. buccinoides* and the other 10 species (Figure S3, Table S14). Comparisons of the genes of 7 molluscan species including *Octopus bimaculoides*, *Crassostrea gigas*, *Mizuhopecten yessoensis*, *Lottia gigantea*, *Pomacea canaliculate*, *Biomphalaria glabrata*, and *Aplysia californica* were summarized in Figure S4. The *P. buccinoides*-unique gene families were annotated to 21 GO terms and 32 KEGG pathways, mainly including cellular process, cellular community, and signal transduction (Fig. 2c, Table S15).

**Fig. 3:**
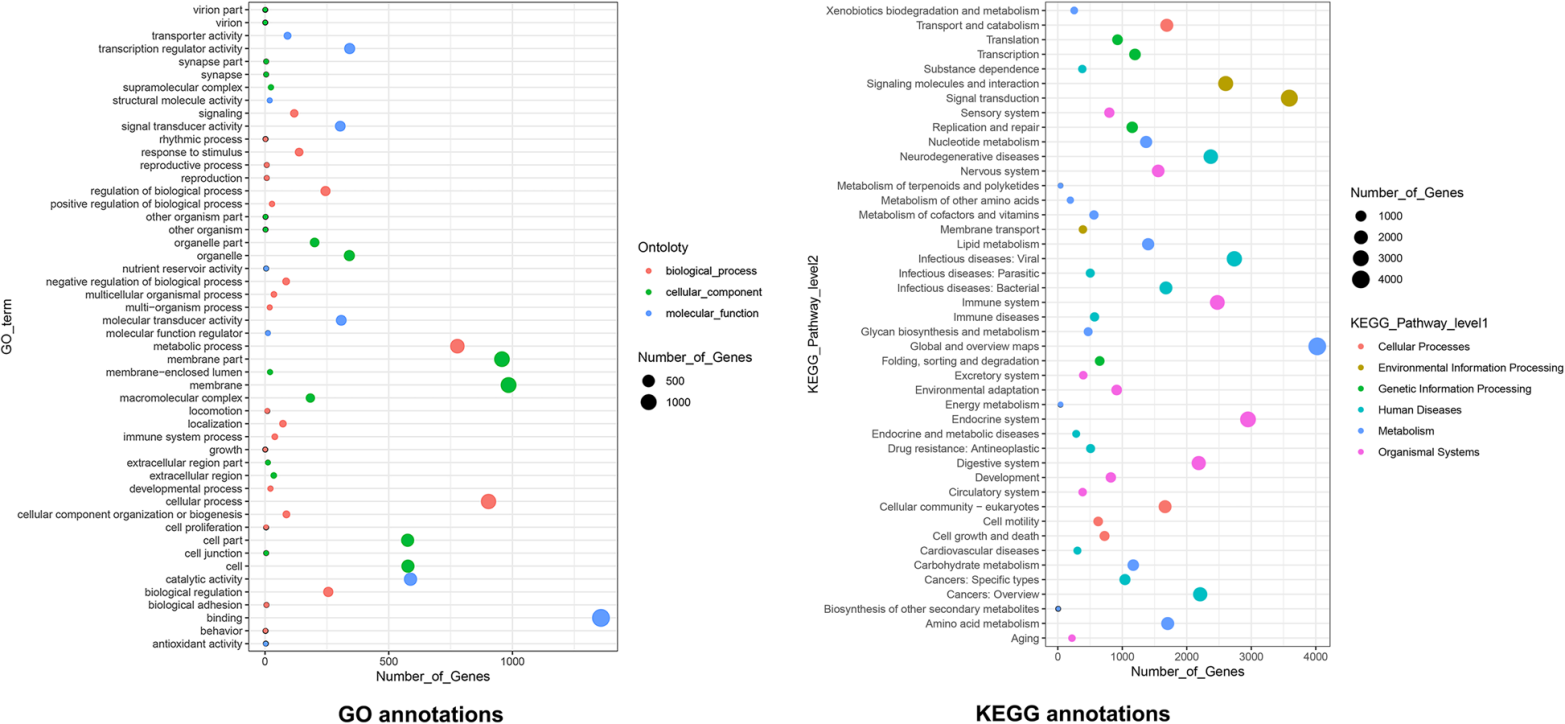
GO and KEGG enrichment analysis of expanded gene families between deep-sea gastropod *P. buccinoides* and shallow sea gastropod *L. gigantea*

Compared with the shallow sea gastropod *L. gigantea*, 562 expanded and 15 contracted gene families were detected in *P. buccinoides.* The expanded genes in *P. buccinoides* were mainly enriched in the items such as environmental adaptation, sensory system, immune system process and antioxidant activity (Fig. 3, Table S16), and most of them were associated with olfactory and chemosensory, glutamate regulation, sulfur metabolism. Besides, GO and KEGG enriched in the contracted gene families of *P. buccinoides* compared with *L. gigantean*, mainly in association with metabolism, catalysis, and transport were showed in Figure S5, Table S17.

A phylogenetic tree was constructed using the single-copy gene families to investigate the phylogenetic evolutionary relationships among *P. buccinoides* and the other species. Phylogenetic analysis suggested that *P. buccinoides* diverged from *Pomacea canaliculata* approximately 218.9 million years ago (Mya). The estimated divergence time between *L. gigantea* and the branch group including *P. buccinoides* was 437.3 Mya. The gastropod showed an estimated divergence time of approximately 498.7 Mya from its sister group bivalve, and the time that cephalopod diverged from other lineages of mollusca was about 550.6 Mya. The mollusca were separated from brachiopoda, annelida and arthropoda about 608.2, 682.3 and 762.4 Mya (Fig. 2b).

### Genomic basics of deep-sea dark, cold and toxic adaptation

**Fig. 4:**
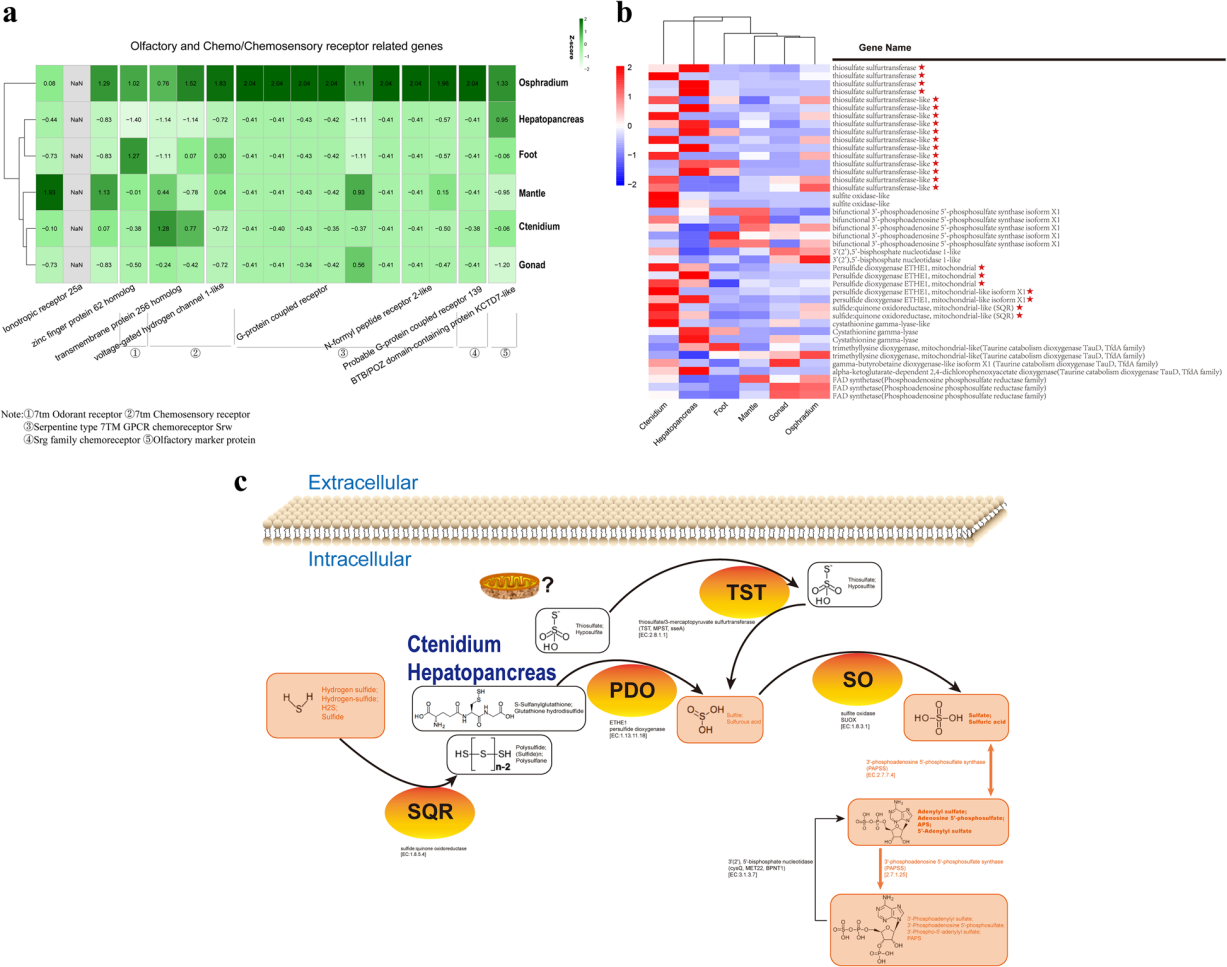
Heat map and hierarchical clustering showing expression of key genes impacting deep-sea environments adaptation and overview of sulfide detoxification in *P. buccinoides*. **a** Expression of olfactory and chemo/chemosensory receptor related genes in six tissues. **b** Expression of sulfur metabolism related genes in different tissues. The genes marked with red stars are important genes which are involved in the sulfide detoxification process. c Overview of sulfide detoxification in *P. buccinoide*. The model of sulfide detoxification of *P. buccinoides* is shown

Results from the genomic comparative analysis showed that the glutamate regulative and related genes including glutamine synthetase, glutamine γ-glutamyltransferase, and γ-aminobutyric acid receptor (GABA(A)) were expanded in *P. buccinoides* genome comparing with that in its relative specie living in shallow water (Fig. 3, Table S16). The olfactory/chemosensory gene families, such as olfactory specific medium-chain acyl CoA synthetase, serpentine type seven transmembrane GPCR chemoreceptor srw, G-protein coupled receptor were also significantly expanded (Fig. 3, Table S16). RNA-seqs of six tissues including hepatopancreas, foot, mantle, ctenidium, gonad and osphradium were also conducted to identify differentially expressed genes (DEGs) corresponding to olfactory and chemo/chemosensory receptor and sulfur metabolism related genes (Fig. 4, Table S18). A total of 17 olfactory/chemosensory genes, including ionotropic receptor 25a, zinc finger protein 62 homolog, transmembrane protein 256 homolog, voltage-gated hydrogen channel 1-like, G-protein coupled receptor, N-formyl peptide receptor 2-like, probable G-protein coupled receptor 139, and BTB/POZ domain containing protein KCTD7-like, were highly expressed in osphradium. These genes were enriched in five protein families including seven transmembrane odorant receptor, seven transmembrane chemosensory receptor, serpentine type seven transmembrane GPCR chemoreceptor srw, srg family chemoreceptor, and olfactory marker protein (Fig. 4a).

### Pathways and networks related to H_2_S detoxification

Compared with shallow sea gastropod *L. gigantea*, the expanded genes in *P. buccinoides* were also primarily in association with sulfotransferase (Fig. 3, Table S16). Furthermore, the mRNA expression patterns of the sulfur metabolism related genes in the tissues of osphradium, hepatopancreas, foot, mantle, ctenidium and gonad were also detected. As shown in Fig. 4b, c and Table S18, the characteristics and expression levels of key sulfur metabolism related genes including sulfite oxidase-like, bifunctional 3'-phosphoadenosine 5'-phosphosulfate synthase isoform X1, 3'(2'),5'-bisphosphate nucleotidase 1-like, persulfide dioxygenase ETHE1 mitochondrial (like), sulfide:quinone oxidoreductase mitochondrial-like (SQR), cystathionine gamma-lyase, and genes in taurine catabolism dioxygenase TauD TfdA, phosphoadenosine phosphosulfate reductase protein families were generally highly expressed in ctenidium and/or hepatopancreas.

**Fig. 5:**
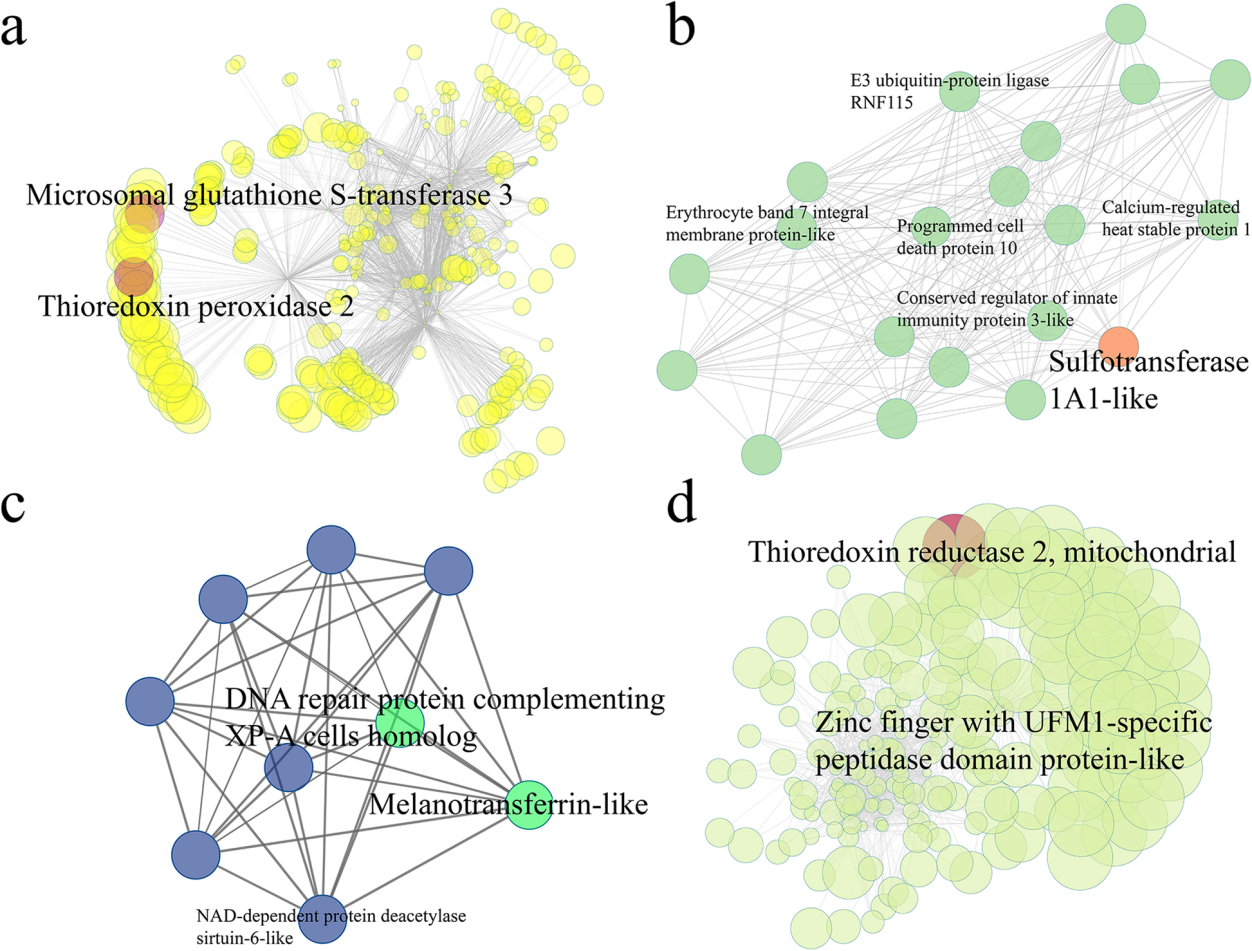
The co-expression network in *P. buccinoides*. Node size represents the intramodular connectivity of a given gene. Gene names are showed for the top hub and key genes. **a** Hepatopancreas-related module (yellow, see Figure S9a). Key genes are labeled in red. **b** Hepatopancreas-related module (lightgreen, see Figure S9a). Sulfur metabolism-related gene is labeled in orange. **c** Hepatopancreas-related module (royalblue, see Figure S9a). Key genes are labeled in green. **d** Ctenidium-related module (greenyellow, see Figure S9b). Key genes are labeled in red

Moreover, the gene co-expression networks based on transcriptome were also constructed to identify hub genes in hepatopancreas and ctenidium tissues. Some key genes relevant to sulfur metabolism and detoxification were detected in the hepatopancreas and ctenidium-related modules. In details, analysis of the yellow module of hepatopancreas showed microsomal glutathione S-transferase 3 and thioredoxin peroxidase 2 were the most important hub genes with the highest intramodular connectivity (Fig. 5a). Sulfotransferase was identified in the network of light green module (Fig. 5b) while Fig. 5c illustrated a group of genes such as melanotransferrin-like, DNA repair protein complementing Xeroderma pigmentosum (XP)-A cells homolog and NAD-dependent protein deacetylase sirtuin-6-like. The zinc finger with UFM1-specific peptidase domain protein-like and thioredoxin reductase 2 were identified in Fig. 5d.

## Discussion

The present study provides the first chromosome-level genome assembly of *P. buccinoides* and the results (Table S8, 9 and Fig. 1d, e) show that the assembled genome of *P. buccinoides* has high integrity and accuracy. The phylogeny of *P. buccinoides* (Fig. 2b) is consistent with previous researches [35–37], which confirms that other species of caenogastropoda are the most closely related species and they evolved together with other gastropod species, which diverged from other molluscan species including cephalopoda and bivalves a long time ago. In gastropod, the caenogastropoda diverged from panpulmonata and euopisthobranchia, and their ancestors diverged from patellogastropoda. Compared with the reported genomes of gastropods, most of which are less than 2 Gb [35, 36, 38–40], the genome size of *P. buccinoides* is relatively large (Table S9). It might be due to the high proportion of repetitive elements (Table S10, 11). For example, the repeats of *Pomacea maculate* (genome size is 432 Mb), *P. canaliculate* (448 Mb), *Lanistes nyassanus* (510 Mb), and *Marisa cornuarietis* (536 Mb) are 20.5–30.8%, and that of *B. glabrata* (916 Mb), *Sinotaia purificata* (984 Mb), *Achatina fulica* (1.85 Gb), *Cepaea nemoralis* (3.5 Gb) and *Oreohelix idahoensis* (5.4 Gb) are 44.8%, 47.93%, 71%, 77% and 85.74%, respectively [36, 39, 41–44]. Overall, the chromosome-level genome assembly of *P. buccinoides* is a valuable resource for studying the adaptation to deep-sea cold seep environments.

Compared with the shallow sea gastropod *L. gigantea*, GO and KEGG enriched in the contracted gene families of *P. buccinoides* are mainly in association with metabolism, catalysis, and transport (Figure S5, Table S17), which is consistent with previous reports that the metabolic rates of deep-sea life are orders of magnitude lower than those of life on Earth's surface [45]. Expansion of gene families play important roles in adaptation to environment including deep sea [46]. It is worth noting that the expanded genes in *P. buccinoides* are mainly associated with glutamate regulation, olfactory and chemosensory and sulfur metabolism (Table S16c).

The glutamate regulative and related genes (glutamine synthetase, glutamine γ-glutamyltransferase, and γ-aminobutyric acid receptor (GABA(A)) are noteworthy since they play important roles in hypoxia acclimation [47]. Glutamate uptake and transport by astrocytes is fundamentally important in the regulation of nervous system function, and hypoxia can suppress the glutamate transport in astrocytes [48]. Neurons and astrocytes can increase the release of glutamate, as well as improve glutamine and/or glutamate utilization to acclimate to the hypoxia condition [49]. Previous studies reveal that glutamate may be a conditionally essential amino acid resulting in enhanced tolerance to hypoxia, cold and amelioration of hypoxia-induced oxidative stress in rats [50, 51]. The γ-aminobutyric acid (GABA) exerts its inhibitory effects by binding to the GABA(A), and cDNA sequences encoding GABA(A) subunit has been cloned in mollusca [52]. GABA causes stress tolerance in plant cells and can be found in most eukaryotic organisms. Under hypoxia/anoxia stress, dual effects of GABA on both pH and TCA pathways play an important role in diminishing injury [53]. Increase of glutamate concentration can promote GABA synthesis in germinated soybean under hypoxia stress [54]. When exposure to hypoxia, GABA is neuroprotective to mature neurons of rat [55]. In the present study, the expansion of the glutamate regulating and related genes implies that the *P. buccinoides* has evolved to be more effective in glutamate production to ensure the physiological activities in a low-oxygen environment, which is of vital importance for its prosperity at the seep habitats. In addition, glutamate regulative genes are also responsive in cold acclimation. It is reported that the release of glutamate evoked by capsaicin is enhanced in spinal dorsal horn slices of repeated cold stress and adjuvant arthritic rats [56], and cold-induced glutamate release in vivo from the magnocellular region of the paraventricular nucleus is involved in ovarian sympathetic activation [57]. Similar to the response to hypoxia, the hosts intend to secrete more glutamate upon cold stress to sustain the physiological activities in mammals [58]. A substantial conversion of glutamate to GABA is proportional to the severity of cold stress and GABA accumulates to a higher extent when exposed to lower temperature. GABA is suspected to involve in tolerance to low temperature [59]. Collectively, results in the present study indicate that the deep-sea snail *P. buccinoides* might be able to produce more glutamate and GABA to adapt to the relatively hypoxic and cold environment at the deep-sea cold seep.

Throughout the animal kingdom, chemical senses are one of the primary means by which organisms make sense of their environment [60]. The size and diversity of chemoreceptors that mediate the transduction of chemical signals can reflect the niche inhabited by the organisms. For example, *Caenorhabditis elegans* requires an abundance of chemoreceptors to navigate and interpret its nutrient-rich living environments, because they spend more foraging time compared to parasitic nematodes [61]. Especially, previous researches show that expansions of G-protein coupled receptor are correlated with environmental adaptations by enabling the evolution of sensory functions in some invertebrate species [62–64]. Therefore, the expansion of olfactory/chemosensory genes in the present study suggests that *P. buccinoides* might rely mainly on the sense of odorants or chemicals for predation and orientation to survive at the dark seep environment. Moreover, the transcriptomic survey revealed that olfactory/chemosensory genes also showed obviously high expression levels in osphradium (Fig. 4a, Table S18). Osphradium is a single or paired chemosensory organ connected with one of the visceral ganglia and situated near the gill of most aquatic molluscs [65, 66]. The olfactory organ is extremely important for molluscan environmental adaptation since it helps them to locate food, nest sites, and escape dangers. For example, nautilus, a well-known “living fossil”, possesses a pair of olfactory organs called rhinophores that are similar to the olfactory organs in octopus and other cephalopods [66]. The rhinophores serve primarily in distance chemoreception during tracking [67]. Odor on a variety of spatial scales is an important information source to nautiluses in their complex coral-reef environment. Olfactory memory for predator and prey is also of great importance to their survival in the wild [68]. The osphradium also possess carrion (or prey) locating function in some gastropods [65]. And, the rhodopsin G-protein coupled receptors are highly expressed in sensory epithelia microdissected from *Aplysia* rhinophore, which are involved in its chemical detection [69]. Thus, the present study indicates that osphradium might be the olfactory/chemosensory organ of *P. buccinoides*. Since the cold seeps are usually characterized as sulfate-rich, hypoxic, dark, organic enrichments such as whale skeletons are released into the ocean, the expansion of olfactory/chemosensory genes in the genome and the relatively high mRNA expression in osphradium suggest that *P. buccinoides* has evolved a highly developed and sensitive olfactory organ comparing with their relative specie living in the shallow water, which might contribute to their orientation, predation to adapt to the cold seep environment.

H_2_S is toxic and creates extreme environmental conditions [70–72]. The toxicity of H_2_S is often exhibited through inhibition of cytochrome c oxidase (COX) in the mitochondrial respiratory chain to inhibit ATP production [73]. Exposure to environmental H_2_S will impact the capability of survival and reproduction of an organism [74]. Deep-sea cold seeps are rich in sulfides and heavy metals [2, 11–13, 75]. The tubeworms, deep-sea mussels, and deep-sea snails have thrived at the cold-seep environment by evolving strategies to acclimate to the toxic conditions [75]. Based on recent studies, three mechanisms of H_2_S tolerance have been revealed in different organisms. First, some organisms can minimize the flux rich in H_2_S into the body [76]. Second, the H_2_S tolerance can be achieved through the modifications of toxicity targets that make them less sensitive to adverse consequences caused by elevated endogenous concentration in the face of continuous influx from the environment [77]. Third, the organisms can tolerate to H_2_S by performing detoxification mediated by a series of enzymes such as SQR, ETHE1 and TST [78, 79]. In the present study, SQR, ETHE1, and thiosulfate sulfurtransferase (TST) were highly expressed in ctenidium and hepatopancreas (Fig. 4b, Table S18), and they are responsible for detoxification of H_2_S [79]. Ctenidium is a respiratory organ found in many molluscs, which is responsible for gas (O_2_ and H_2_S) exchange [80]. Hepatopancreas is regarded as a critical organ for metabolism and detoxification in molluscs [81]. Therefore, the current results suggest that *P. buccinoides* might have the strong capability in tolerating sulfide by conducting H_2_S detoxification, and its ctenidium and hepatopancreas are critical tissues for such process. In addition, glutathione S-transferase is a well-known antioxidant and detoxification enzyme, and it contribute to the metabolism of drugs, pesticides and other xenobiotics [82, 83]. The glutathione S-transferase and thioredoxin peroxidases also take part in response to onslaught of oxidants and function in maintaining efficient antioxidant defense in invertebrates [84–86]. In the present study, as the hub genes of hepatopancreas and ctenidium-related modules in gene co-expression networks (Fig. 5), microsomal glutathione S-transferase 3 and thioredoxin peroxidase 2 are presumed to be key genes of antioxidant and detoxification in *P. buccinoides*. Besides, sulfotransferase in the network utilizes 3′-phospho-5′-adenylyl sulfate (PAPS) as a sulfonate donor [87] to participate in sulfur metabolism, while the zinc finger with UFM1-specific peptidase domain protein-like (deubiquitylating enzyme) and thioredoxin reductase 2 are speculated to play important roles in DNA damage responses and repair [88]. In summary, it is quite possible that *P. buccinoides* adapts to the sulfide-rich cold seep environment by enhancing the H_2_S detoxification activities in ctenidium and hepatopancreas. It has been reported that there are no symbiotic bacteria in ctenidium of *P. buccinoides* [32], however, whether the sulfide detoxification is performed by endosymbiotic bacteria in other tissues deserves further investigation in the future.

## Conclusions

The first chromosome-level genome assembly of the deep-sea snail *P. buccinoides* was constructed in the present study. The genome size of *P. buccinoides* is relatively large (about 2.1 Gb, scaffold N50 = 60.0 Mb) compared with the other known snail genomes, which might be due to the high proportion of repetitive elements. The glutamate regulative and related gene family was found to be expanded, which might contribute to the acclimation to hypoxia and coldness. The relatively high mRNA expression of the olfactory and chemosensory related genes in osphradium indicates that *P. buccinoides* might have evolved a highly developed and sensitive olfactory organ for its orientation and predation. More importantly, results of the transcriptomic and network analysis showed that *P. buccinoides* might have evolved sulfite tolerance mechanism by performing H_2_S detoxification in ctenidium and hepatopancreas. The present study provides insights into the mechanisms of adaptation of gastropod to the dark, hypoxic and H_2_S-rich environment in the deep-sea cold seeps.

## Methods

### Sample collection and sequencing

The deep-sea snails *P. buccinoides* (Fig. 1a) were collected at cold seep site F (22°06′N, 119°17′E), which was located on the continental slope of the South Sea of China during the expedition cruise of the R/V Kexue in 2018 (Fig. 1b). These snails were found 1,119 m beneath the sea surface with temperatures of 3.35–3.89 °C, salinity of 34.53–35.54 psu, and dissolved oxygen of 3.01–3.18 mg/L. In the bottom water of the chemosynthetic communities, the H_2_S level increased remarkably and the highest H_2_S level (~ 1940 μM) among all seawater samples was in the bottom water above the reduced sediments [22]. The samples were preserved at -80 ℃. All of the experiments were performed following the animal ethics guidelines approved by the Ethics Committee of Dalian Ocean University.

Genomic DNA was extracted from hepatopancreas and muscle of snails using the modified phenol/chloroform method [89]. One sequencing library with insert size of 350 bp was generated using Truseq Nano DNA HT Sample Preparation Kit (Illumina, San Diego, CA, USA) following manufacturer’s recommendations. PacBio library with insert size of 20 kb was constructed using PacBio single molecule, real-time long reads sequencing technology (SMRT) SMRTbell Template Prep Kits (PacBio, Menlo Park, CA, USA). One short insert size (350 bp) library was sequenced on NovaSeq 6000 platform (Illumina, San Diego, CA, USA) using whole-genome shotgun sequencing (WGS) strategy. The raw data were generated and filtered by SOAPFILTER v2.2, a software in the SOAPdenovo package [90]. The long insert size (20 kb) library was sequenced on PacBio Sequel instrument (PacBio, Menlo Park, CA, USA) to obtain long reads (polymerase reads) data. After removing the adapters, the polymerase reads were partitioned to form subreads (Pacific Biosciences Terminology).

A total of 1000 μL (10.3 ng/μL) hepatopancreas sample of *P. buccinoides* from the same collection lot was treated [91] and the Hi‐C libraries were constructed with NEBNext Ultra II DNA library Prep Kit for Illumina (NEB, Ipswich, MA, USA). The target fragments were captured with Dynabeads MyOne Streptavidin C1 (Thermo Fisher Scientific, Inc., Waltham, MA, USA). After that, the chimeric fragments were amplified with NEBNext Ultra II DNA library Prep Kit (NEB, Ipswich, MA, USA). Two replicates of libraries were generated and the libraries were sequenced on Illumina Novaseq 6000 instrument (Illumina, San Diego, CA, USA).

To perform the single-molecule long-read transcriptome sequencing with SMRT, the hepatopancreas, foot, mantle, ctenidium, gonad, and osphradium tissues were harvested. The sample is precious in the present study and three biological and technical replicates should be performed generally. RNA from different tissue samples was isolated using Trizol reagent (Sangon, Shanghai, China). By using NEBNext Ultra RNA Library Prep Kit for Illumina (NEB, MA, USA), short read RNA-Seq libraries were prepared and then sequenced on NovaSeq 6000 platform (Illumina, San Diego, CA, USA). With the Clontech SMARTer PCR cDNA Synthesis Kit (Clontech, CA, USA), one SMART bell library was constructed. The SMRT sequencing was performed on Pacific Bioscience Sequel System (Pacific Biosciences, CA, USA).

### Assemblies of genome and transcriptome

All cleaned reads from short insert library were assembled using PLATANUS v1.2.4 [33] with parameter “-k 27” to obtain a de Bruijn graph assembly. Subsequently, the DBG2OLC was employed to align the de Bruijn graph assembly upon the PacBio reads for further construction of contigs [34]. Three rounds of mapping were performed with MINIMAP v2.1 [92] and polished with RACON v1.3.1 [93] to construct consensus contigs. Then, BWA v0.7.15 [94] and PILON v1.22 [95] were used to polish the assembly one round with 350 bp library Illumina paired-end reads. Completeness of the final assembly at contig level was assessed using BUSCO v3.1.0 [96]. The mollusca_odb10 [97] orthologues gene set was used as the BUSCO reference.

The raw data generated from Hi‐C library were filtered with TRIMMOMATIC v0.39 [98], and the clean data were aligned against the draft genome using JUICER v1.6.2 [99] with the default parameters. The Hi-C contacts without duplicates were used to assist genomic assembly by 3D-DNA v180114 [100]. The heatmap of chromosome interactions was constructed with 3D-DNA v180114 [100] to visualize the contact intensity among Chrs. The scaffolds were assembled and the obtained genome at contig-level was located onto the Chrs. By using JUICEBOX v1.8.8 [101], the Hi-C contact map was visualized and the extensive manual curation was performed to ensure the scaffolds within the same pseudo-chromosomal linkage group to meet the Hi-C linkage characteristics. The clustered contigs and mis-joins were ordered, oriented and fixed.

By using SMRTLINK v6.0 software (Pacific Biosciences, CA, USA) (https://www.pacb.com/support/software-downloads), the non-chimeric circular consensus sequences (CCSs) were generated from subread BAM files and a ccs.bam file was obtained. By performing the isoform level Iterative Clustering for Error Correction (ICE), consensus isoforms were identified from Full-length non-chimeric (FLNC) then and they were further polished with QUIVER v2.2.2 [102]. To correct nucleotide indels and mismatches in consensus reads resulting in corrected isoforms, the Illumina RNA-Seq data with same samples were used by LoRDEC v0.7 [103]. By using CD-HIT v4.6.8 [104], the redundancies in corrected consensus reads were removed and final non-redundancy transcripts for the subsequent analysis were obtained.

By using SOAPnuke v1.5.6 [105], the low-quality reads (quality score ≤ 20) were removed. The clean transcriptome reads were assembled using TRINITY v2.5.1 [106]. By using BOWTIE v1.1.1 [107], sequences obtained by Illumina-Seq were aligned to the transcript obtained by SMRT sequencing, regarded as a reference. The gene expression levels of each sample were estimated with Expectation–Maximization (RSEM) v1.3.0 [108]. By using the GOseq R package v1.10.0 [109], GO enrichment analysis of DEGs were performed. GO terms of DEGs with corrected *P*-value < 0.05 were considered enriched significantly. The statistical significant enrichments of DEGs in KEGG pathways were determined by KOBAS v3.0 [110] with the *P*-value < 0.05.

### Structure and functional annotation

Prior to gene prediction using the assembled genome, de novo and homology-based prediction were used to annotate repeat elements. The local de novo repeat reference library was generated using LTR FINDER v1.0.6 [111], REPEATMASKER v4.0.6 [112] and REPEATMODELER v1.08 [113]. Subsequently, the assembled genome was aligned against this reference to produce the de novo predicted repeat elements. For the homology-based prediction, REPEATPROTEINMASK v4.06 [112], REPEATMASKER v4.0.6 [112] and TANDEM REPEATS FINDER (TRF) v4.07 [114] were run to identify, classify and mask repeats with REPBASE v21.01 [115] in the *P. buccinoides* genome. Finally, the non-redundant results were generated by integrating the data from two predictions.

The assembly was annotated with three different strategies. AUGUSTUS v2.5 [116], GENSCAN v1.0 [117] and SNAP v2.0 [118] were employed for the first ab initio gene prediction method and the genome assembly was masked to exclude the repetitive elements firstly. Homologous-gene-based annotation was the second method. The protein sequences of California sea hare (*A. californica*), a freshwater snail (*B. glabrata*) [119], roundworm (*C. elegans*) [120], Eastern oyster (*Crassostrea virginica*) [121], Pacific oyster (*C. gigas*) [122], fruit fly (*Drosophila melanogaster*) [123], owl limpet (*L. gigantea*) [124], Yesso scallop (*M. yessoensis*) [125], California two-spot octopus *O. bimaculoides* [126] and golden apple snail *P. canaliculata* [35] were downloaded from the NCBI database. The TBLASTN in Basic local alignment search tool (BLAST) v2.2.26 program [127] was used to search for best-hit alignments of these proteins in the assembled *P. buccinoides* genome with E-value cutoff of 10^–5^. Then the potential gene structure of each best-hit alignment was identified with GENEWISE v2.4.1 [128]. The transcriptomic data generated from mantle, ctenidium and 6 tissues were mapped onto the assembly to aid gene annotation. The final resultant was obtained using MAKER v2.31.8 [129].

By using BLAST v2.2.26 [127], the functional motifs and domains were identified by searching the predicted genes of *P. buccinoides* in NCBI non-redundant protein sequences (NCBI-Nr) [130], Swiss-Prot [131], Interpro [132], Clusters of Orthologous Groups (COG) [133], TrEMBL, KEGG [134] and GO [135] public functional databases. By using BLAST software v2.7.1 [127] under a threshold E-value ≤ 1e-5, corrected isoforms of long read transcripts were searched against NCBI-Nr [130], NCBI-Nt, Swiss-Prot [131], KOG/COG [136, 137] and KEGG v2015_10_10 [134]. The Protein family (Pfam) database [138] was searched by HMMER v3.1 [139], and the Pfam accession numbers were converted to GO terms by using ‘pfam2go’ mapping [135].

### Phylogenetic analysis of the genome

The complete gene set of *P. buccinoides* and other 10 representative species including *A. californica* (GCF_000002075.1), *B. glabrata* (GCF_000457365.1) [140], *C. gigas* (GCF_000297895.1) [122], *D. melanogaster* (GCF_000001215.4) [123], *Helobdella robusta* (GCF_000326865.1) [124], *Lingula anatina* (GCF_001039355.2) [37], *L. gigantea* (GCF_000327385.1) [124], *M. yessoensis* (GCF_002113885.1) [125], *O. bimaculoides* (GCF_001194135.1) [126], and *P. canaliculata* (GCF_003073045.1) [35] were downloaded from NCBI. To check the homology and generate a sequence similarity matrix, the whole-genome gene sets were aligned with BLAST v2.6.0 [127]. ORTHOMCL v1.4 [141] with 1.5 inflation index was employed to distinguish gene families from the sequence similarity matrix. MUSCLE v3.8.31 [142] was used to determine homologous genes and identified single-copy orthologs. By using PHYML v3.0 [143], the phylogenetic topology with the maximum likelihood (ML) method was estimated with gamma distribution across aligned sites and HKY85 substitution model to construct the phylogenetic tree. *D. melanogaster* was used as the outgroup. To estimate divergence times among the *P. buccinoides* and the other molluscan species, the MCMCTREE in PAML v4.4 [144] was employed. The neutral evolutionary rate and species divergence time were estimated by adopting the Bayesian molecular dating [145]. Five reference divergence time points retrieved from the TimeTree database [146] were used to calibrate the phylogenetic tree [147–151].

### Expansion and contraction of gene families

The program CAFÉ v2.1 [152] was adopted by determining the evolutionary dynamics of gene families to identify gene family changes between the deep-sea gastropod *P. buccinoides* and shallow sea gastropod *L. gigantea*, especially expansion and contraction of gene ortholog clusters. The gene families presented uniquely in *P. buccinoides* were also screened. Venn diagram was drawn with VENNPAINTER v1.2.0 [153]. KEGG and GO analysis of the gene families exclusively presented and specifically expanded and contracted in the *P. buccinoides* were conducted as that in functional annotation [127, 134, 135].

### Network and other analysis

By using 6 transcriptome datasets of different tissues (hepatopancreas, foot, mantle, ctenidium, gonad, and osphradium) with WGCNA v1.70–3 [154], co-expression gene networks were constructed. Cytoscape software v3.9.1 was used to visualize the networks [155]. Block-wise network construction and consensus module detection methods were adopted, with the parameters of soft-thresholding power = 14, maximum block size = 2000 and minimum module size = 30. Module eigengene *E* was calculated to identify the tissue-related modules. The hub genes in a given module was measured by its connection strength with other genes in the module, and was determined by intramodular connectivity [154].

By using MIcroSAtellite identification tool (MISA) v1.0 [156], the microsatellites as well as compound microsatellites were identified and localized. The Animal Transcription Factor Data Base v2.0 (animalTFDB) [157] were used to predict the transcription factors. The analysis methods of long non-coding RNA (lncRNA) were performed with Coding Potential Calculator (CPC) v0.9 [158], Coding-Non-Coding Index (CNCI) v2.0 [159], predictor of long non-coding RNAs and messenger RNAs based on an improved k-mer scheme (PLEK) v1.2 [160] and Pfam v1.6 [138].

### Supplementary Information

**Additional file 1: Figure S1.** Divergence distribution of transposable elements (TEs) in the *P. buccinoides* genome. **Figure S2.** Chromosomal syntenic relationships. **Figure S3.** Distribution of genes in 11 different species. **Figure S4.** Venn diagram of gene families specific to *P. buccinoides*. **Figure S5.** GO and KEGG enrichment analysis of contracted gene families between deep-sea gastropod *P. buccinoides* and shallow sea gastropod *L. gigantea*. **Figure S6.** Length distribution of unigenes in transcriptome. **Figure S7.** Length distribution of coding DNA sequence (CDS) in transcriptome. **Figure S8.** Expression of sulfur metabolism related genes in different tissues. **Figure S9.** Module eigengene *E* of gene co-expression networks. **Figure S10.** Gene dendrograms and module colors of gene co-expression networks. **Figure S11.** The distribution of transcript length of lncRNAs and mRNAs. **Figure S12.** The distribution of SSR motifs in transcriptome. **Figure S13.** The numbers of transcription factors involved in the top transcription factor families of transcriptome. **Table S1.** Illumina statistics of the genome sequencing data of *P. buccinoides*. **Table S2.** PacBio statistics of the genome sequencing data of *P. buccinoides*. **Table S3.** Hi-C statistics of the genome sequencing data of *P. buccinoides*. **Table S4a.** Statistics of *P. buccinoides* Illumina transcriptome reads (Raw data). **Table S4b.** Statistics of *P. buccinoides* Illumina transcriptome reads (Clean data). **Table S5.** Statistics of *P. buccinoides *Iso transcriptome reads. **Table S6.** Transcriptome sequencing data of *P. buccinoides* (for aiding gene annotation). **Table S7.** Summary statistics of the genome sequencing data of *P. buccinoides*. **Table S8.** Contig assembly of the *P. buccinoides* genome using Illumina and PacBio reads. Related to Figure 1e. **Table S9.** Summary statistics of the *P. buccinoides* chromosomal-level genome assembly. Related to Figure 1d, e. **Table S10.** Prediction of repeat elements in the *P. buccinoides* genome. Related to Figure 1c, S1. **Table S11.** Categories of repeat elements predicted in the *P. buccinoides* genome. Related to Figure 1c, S1. **Table S12.** Prediction of gene structure in *P. buccinoides* genomes. Related to Figure 1e. **Table S13.** Functional annotation of the predicted protein-coding genes in the *P. buccinoides*. Related to Figure 2a. **Table S14.** Statistics of gene families in 11 examined species.

**Additional file 2: Table S15a.** GO enrichment of unique gene families in *P. buccinoides* compared with seven other molluscan species. Related to Figure 2c. **Table S15b.** KEGG enrichment of unique gene families in *P. buccinoides* compared with seven other molluscan species. Related to Figure 2c.

**Additional file 3: Table S16a.** Enriched GO terms of expanded genes in the deep-sea gastropod *P. buccinoides* compared to shallow sea *L. gigantea*. Related to Figure 3. **Table S16b.** Enriched KEGG pathways of expanded genes in the deep-sea gastropod *P. buccinoides* compared to shallow sea *L. gigantea*. Related to Figure 3. **Table S16c.** Targeted expanded genes in the deep-sea gastropod *P. buccinoides* compared to shallow sea *L. gigantea*. Related to Figure 3.

**Additional file 4: Table S17a.** Enriched GO terms of contracted genes in the deep-sea gastropod *P. buccinoides* compared to shallow sea *L. gigantea*. Related to Figure S5. **Table S17b.** Enriched KEGG pathways of contracted genes in the deep-sea gastropod *P. buccinoides* compared to shallow sea *L. gigantea*. Related to Figure S5.

**Additional file 5: Table S18.** RNA-seq differentially expressed genes (DEGs) in different tissues of *P. buccinoides*. Related to Figure 4, 5.

**Additional file 6: Table S19.** SSRs in RNA-seq of *P. buccinoides*. Related to Figure S12.

**Additional file 7: Table S20a.** Transcription factor in RNA-seq of *P. buccinoides*. Related to Figure S13. **Table S20b.** Statistics of transcription factor in *P. buccinoides* RNA-seq. Related to Figure S13.

**Additional file 8: Table S21.** Functional annotation of *P. buccinoides*.

## Data Availability

The datasets generated during the current study are available in the NCBI under accession number PRJNA700822 (https://www.ncbi.nlm.nih.gov/bioproject/PRJNA700822).
