# Supplementary material for: Chromosome-level genome assembly of the deep-sea snail Phymorhynchus buccinoides provides insights into the adaptation to the cold seep habitat
Source: BMC Genomics. 2023 Nov 10;24:679. doi: 10.1186/s12864-023-09760-0 (PMC10638732; doi:10.1186/s12864-023-09760-0)
Supplement: Supplementary file 1 — Additional file 1: Figure S1. Divergence distribution of transposable elements (TEs) in the P. buccinoides genome. Figure S2. Chromosomal syntenic relationships. Figure S3. Distribution of genes in 11 different species. Figure S4. Venn diagram of gene families specific to P. buccinoides. Figure S5. GO and KEGG enrichment analysis of contracted gene families between deep-sea gastropod P. buccinoides and shallow sea gastropod L. gigantea. Figure S6. Length distribution of unigenes in transcriptome. Figure S7. Length distribution of coding DNA sequence (CDS) in transcriptome. Figure S8. Expression of sulfur metabolism related genes in different tissues. Figure S9. Module eigengene E of gene co-expression networks. Figure S10. Gene dendrograms and module colors of gene co-expression networks. Figure S11. The distribution of transcript length of lncRNAs and mRNAs. Figure S12. The distribution of SSR motifs in transcriptome. Figure S13. The numbers of transcription factors involved in the top transcription factor families of transcriptome. Table S1. Illumina statistics of the genome sequencing data of P. buccinoides. Table S2. PacBio statistics of the genome sequencing data of P. buccinoides. Table S3. Hi-C statistics of the genome sequencing data of P. buccinoides. Table S4a. Statistics of P. buccinoides Illumina transcriptome reads (Raw data). Table S4b. Statistics of P. buccinoides Illumina transcriptome reads (Clean data). Table S5. Statistics of P. buccinoides Iso transcriptome reads. Table S6. Transcriptome sequencing data of P. buccinoides (for aiding gene annotation). Table S7. Summary statistics of the genome sequencing data of P. buccinoides. Table S8. Contig assembly of the P. buccinoides genome using Illumina and PacBio reads. Related to Figure 1e. Table S9. Summary statistics of the P. buccinoides chromosomal-level genome assembly. Related to Figure 1d, e. Table S10. Prediction of repeat elements in the P. buccinoides genome. Related to Figure 1c, S1. Table S [file 12864_2023_9760_MOESM1_ESM.docx]

**Supplemental Information for:**

**Chromosome-level genome assembly of the deep-sea snail *Phymorhynchus buccinoides* provides insights into the adaptation to the cold seep habitat**

**Table of Contents:**

[Supplementary Figures 1](#_Toc140761058)

[Figure S1. Divergence distribution of transposable elements (TEs) in the *P. buccinoides* genome. 1](#_Toc140761059)

[Figure S2. Chromosomal syntenic relationships. 1](#_Toc140761060)

[Figure S3. Distribution of genes in 11 different species. 2](#_Toc140761061)

[Figure S4. Venn diagram of gene families specific to *P. buccinoides*. 3](#_Toc140761062)

[Figure S5. GO and KEGG enrichment analysis of contracted gene families between deep-sea gastropod *P. buccinoides* and shallow sea gastropod *L. gigantea*. 3](#_Toc140761063)

[Figure S6. Length distribution of unigenes in transcriptome. 4](#_Toc140761064)

[Figure S7. Length distribution of coding DNA sequence (CDS) in transcriptome. 4](#_Toc140761065)

[Figure S8. Expression of sulfur metabolism related genes in different tissues. 5](#_Toc140761066)

[Figure S9. Module eigengene *E* of gene co-expression networks. 6](#_Toc140761067)

[Figure S10. Gene dendrograms and module colors of gene co-expression networks. 6](#_Toc140761068)

[Figure S11. The distribution of transcript length of lncRNAs and mRNAs. 8](#_Toc140761069)

[Figure S12. The distribution of SSR motifs in transcriptome. 8](#_Toc140761070)

[Figure S13. The numbers of transcription factors involved in the top transcription factor families of transcriptome. 9](#_Toc140761071)

[Supplementary Tables 10](#_Toc140761072)

[Table S1. Illumina statistics of the genome sequencing data of *P. buccinoides*. 10](#_Toc140761073)

[Table S2. PacBio statistics of the genome sequencing data of *P. buccinoides*. 10](#_Toc140761074)

[Table S3. Hi-C statistics of the genome sequencing data of *P. buccinoides*. 11](#_Toc140761075)

[Table S4a. Statistics of *P. buccinoides* Illumina transcriptome reads (Raw data). 11](#_Toc140761076)

[Table S4b. Statistics of *P. buccinoides* Illumina transcriptome reads (Clean data). 11](#_Toc140761077)

[Table S5. Statistics of *P. buccinoides* Iso transcriptome reads. 12](#_Toc140761078)

[Table S6. Transcriptome sequencing data of *P. buccinoides* (for aiding gene annotation). 13](#_Toc140761079)

[Table S7. Summary statistics of the genome sequencing data of *P. buccinoides*. 13](#_Toc140761080)

[Table S8. Contig assembly of the *P. buccinoides* genome using Illumina and PacBio reads. Related to Figure 1e. 13](#_Toc140761081)

[Table S9. Summary statistics of the *P. buccinoides* chromosomal-level genome assembly. Related to Figure 1d, e. 14](#_Toc140761082)

[Table S10. Prediction of repeat elements in the *P. buccinoides* genome. Related to Figure 1c, S1. 14](#_Toc140761083)

[Table S11. Categories of repeat elements predicted in the *P. buccinoides* genome. Related to Figure 1c, S1. 15](#_Toc140761084)

[Table S12. Prediction of gene structure in *P. buccinoides* genomes. Related to Figure 1e. 15](#_Toc140761085)

[Table S13. Functional annotation of the predicted protein-coding genes in the *P. buccinoides*. Related to Figure 2a. 16](#_Toc140761086)

[Table S14. Statistics of gene families in 11 examined species. 16](#_Toc140761087)

[Table S15a. GO enrichment of unique gene families in *P. buccinoides* compared with seven other molluscan species. Related to Figure 2c. 17](#_Toc140761088)

[Table S15b. KEGG enrichment of unique gene families in *P. buccinoides* compared with seven other molluscan species. Related to Figure 2c. 17](#_Toc140761089)

[Table S16a. Enriched GO terms of expanded genes in the deep-sea gastropod *P. buccinoides* compared to shallow sea *L. gigantea*. Related to Figure 3. 17](#_Toc140761090)

[Table S16b. Enriched KEGG pathways of expanded genes in the deep-sea gastropod *P. buccinoides* compared to shallow sea *L. gigantea*. Related to Figure 3. 17](#_Toc140761091)

[Table S16c. Targeted expanded genes in the deep-sea gastropod *P. buccinoides* compared to shallow sea *L. gigantea*. Related to Figure 3. 17](#_Toc140761092)

[Table S17a. Enriched GO terms of contracted genes in the deep-sea gastropod *P. buccinoides* compared to shallow sea *L. gigantea*. Related to Figure S5. 17](#_Toc140761093)

[Table S17b. Enriched KEGG pathways of contracted genes in the deep-sea gastropod *P. buccinoides* compared to shallow sea *L. gigantea*. Related to Figure S5. 17](#_Toc140761094)

[Table S18. RNA-seq differentially expressed genes (DEGs) in different tissues of *P. buccinoides*. Related to Figure 4, 5. 17](#_Toc140761095)

[Table S19. SSRs in RNA-seq of *P. buccinoides*. Related to Figure S12. 17](#_Toc140761096)

[Table S20a. Transcription factor in RNA-seq of *P. buccinoides*. Related to Figure S13. 17](#_Toc140761097)

[Table S20b. Statistics of transcription factor in *P. buccinoides* RNA-seq. Related to Figure S13. 17](#_Toc140761098)

[Table S21. Functional annotation of *P. buccinoides*. 17](#_Toc140761099)

The source data of Supplementary Tables S15, S16, S17, S18, S19, S20 and S21 are provided as excel files.

# Supplementary Figures


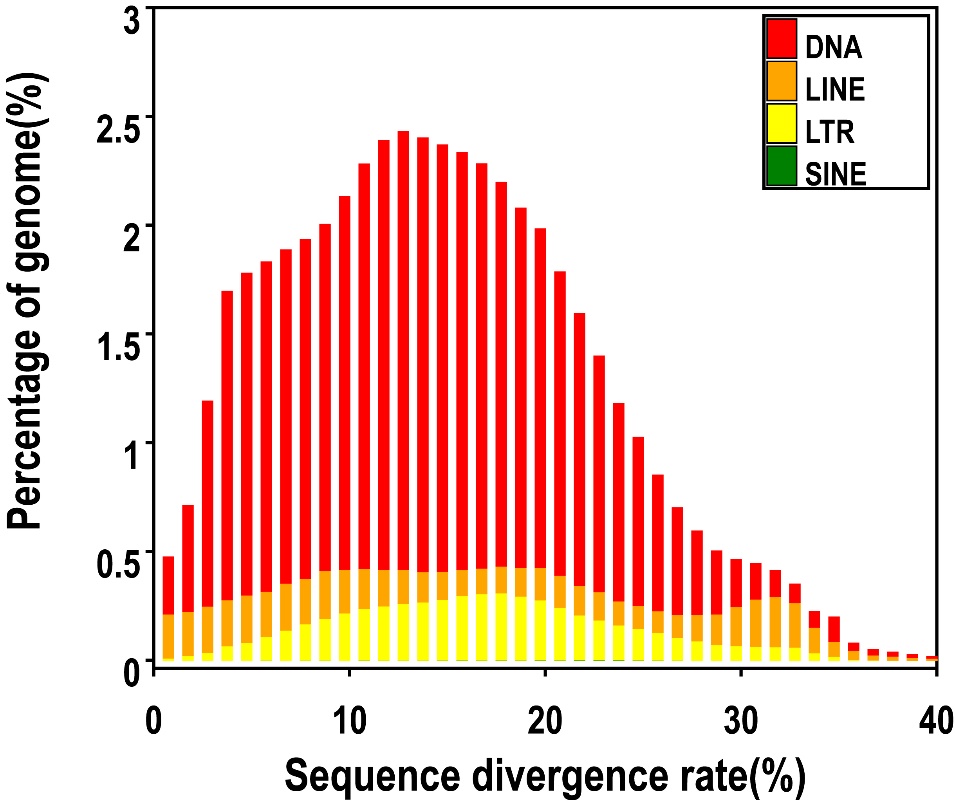


## Figure S1. Divergence distribution of transposable elements (TEs) in the *P. buccinoides* genome.

Homology-based prediction. The distribution of sequence divergence rates of TEs as percentages of the genome size was shown. The y-axis shows the percentage of the genome that is annotated as TEs (TE contents). The x-axis shows sequence divergence rate. DNA transposon shown as DNA is indicated with red color, long interspersed nuclear element (LINE) is indicated with orange color, long terminal repeat (LTR) is indicated with yellow color, and short interspersed nuclear element (SINE) is indicated with green color.


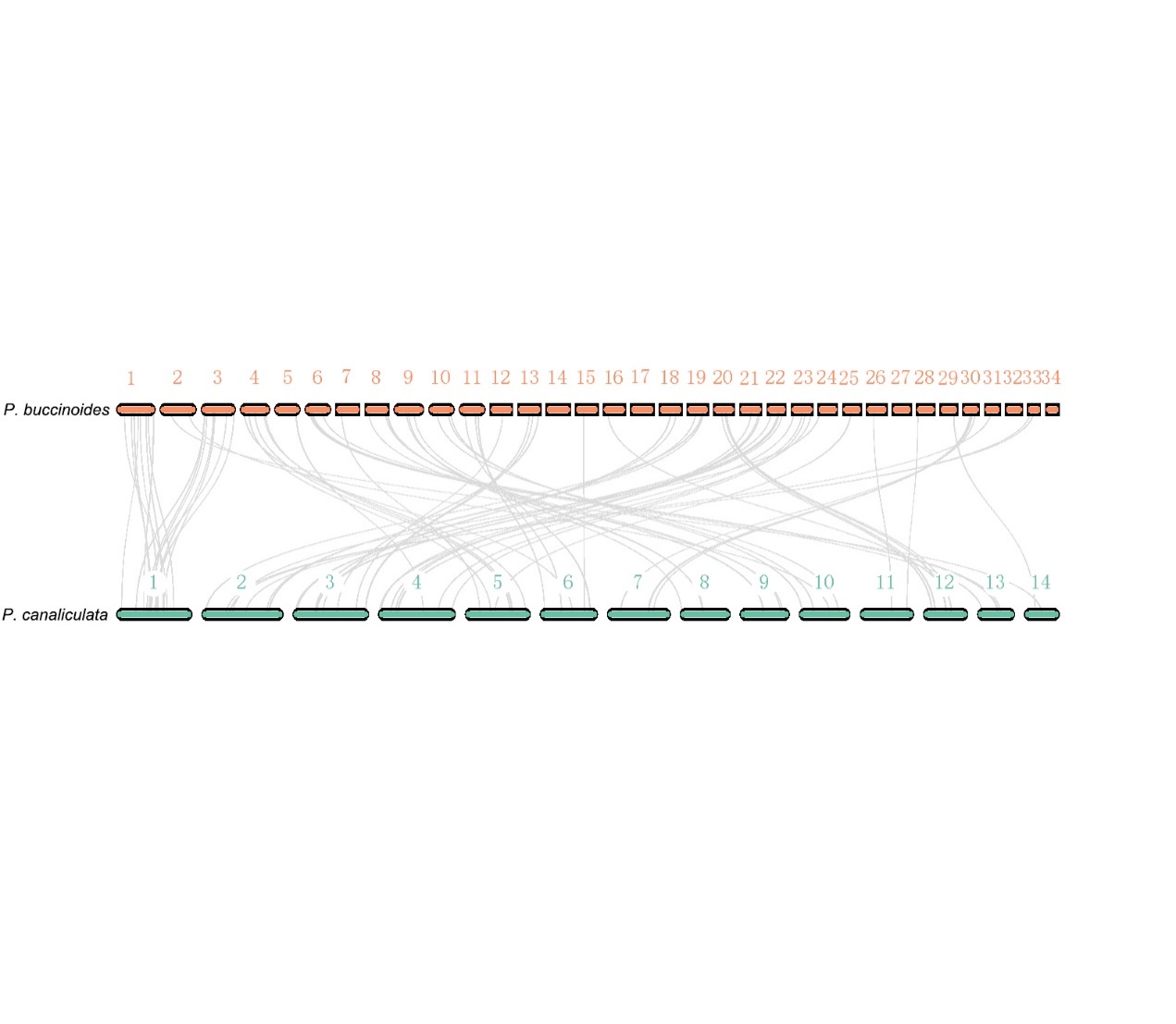


## Figure S2. Chromosomal syntenic relationships.

**(a) Chromosomal Syntenic Relationships between *P. buccinoides* and** ***P. canaliculata*.** Syntenic regions are connected with lines. There are numerous links between chromosomes of *P. buccinoides* and chromosomes of *P. canaliculate*.


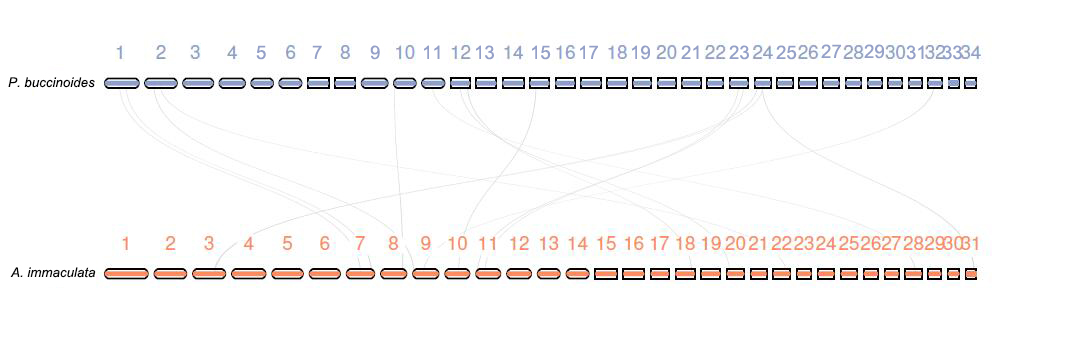


**(b) Chromosomal syntenic relationships between *P. buccinoides* and *A. immaculata*.**


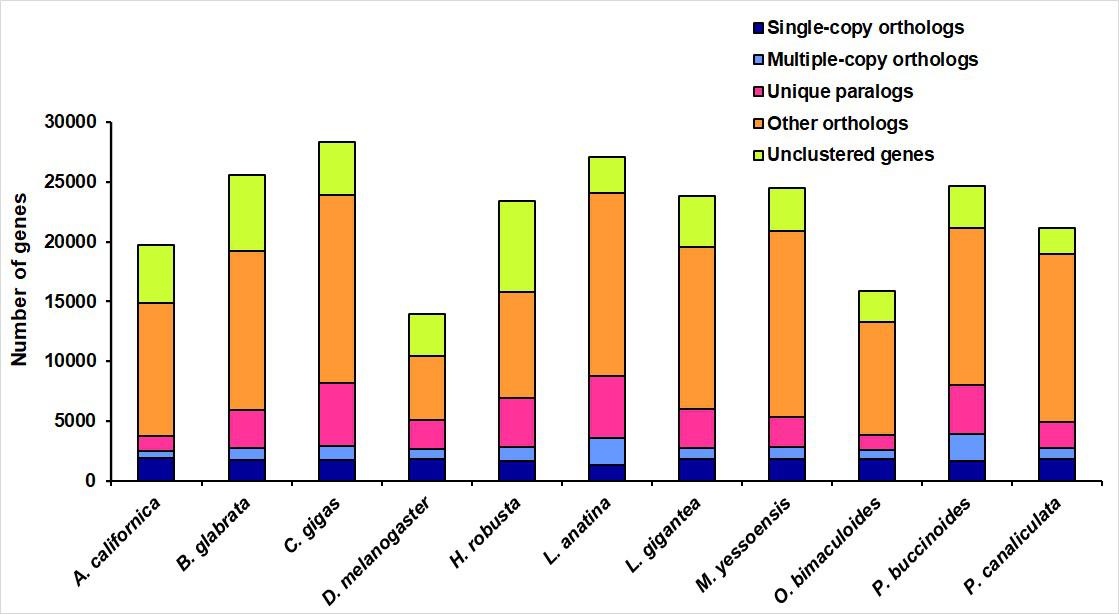


## Figure S3. Distribution of genes in 11 different species.

Gene number distributed in five type of gene families in *P. buccinoides* and other species. Blue indicates single-copy orthologs, and single-copy orthologs include the common orthologs with one copy in the species. Sky blue indicates multiple-copy orthologs, and multiple-copy orthologs include the common orthologs with different copies in the species. Pink indicates unique genes, and unique paralogs include the genes families only existed in one specie. Dark yellow indicates other orthologs, and other orthologs include the orthologs with different copies in more than two but not all species. Unclustered genes include the genes that cannot be assigned into gene families.

**
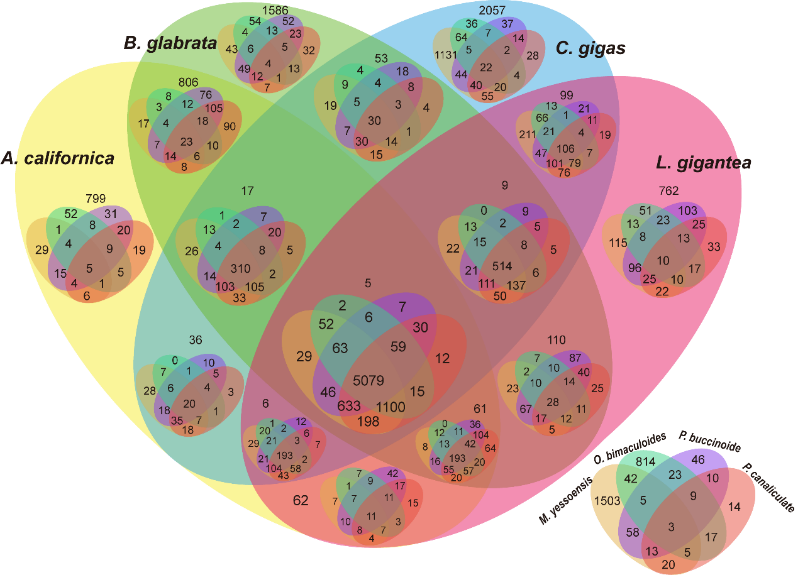
**

## Figure S4. Venn diagram of gene families specific to *P. buccinoides*.

As shown in the legend at the bottom right, numbers of shared genes are in the overlapping areas between different species. Therefore, compared with the 7 molluscan species including *O. bimaculoides*, *C. gigas*, *M. yessoensis*, *L. gigantea*, *P. canaliculate*, *B. glabrata* and *A. californica*, a total of seven gene families are presented uniquely in *P. buccinoides*.


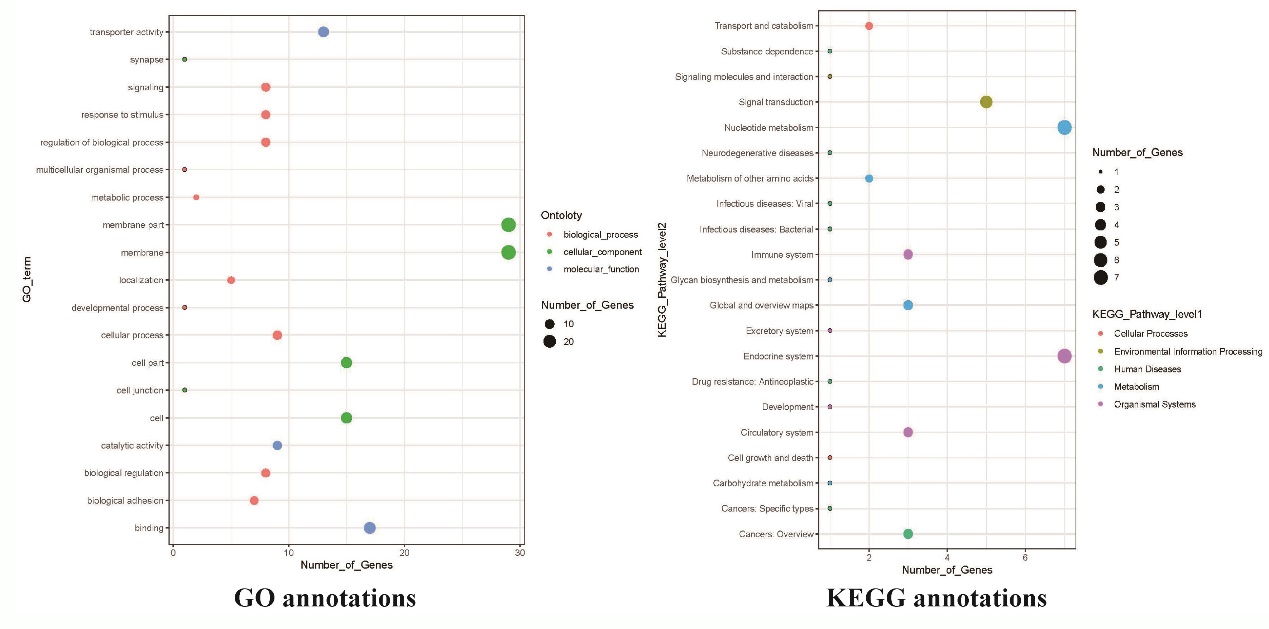


## Figure S5. GO and KEGG enrichment analysis of contracted gene families between deep-sea gastropod *P. buccinoides* and shallow sea gastropod *L. gigantea*.

The x-axis shows the number of genes and the y-axis shows the annotation terms. Different sizes and colors of bubbles exhibit different number and terms.


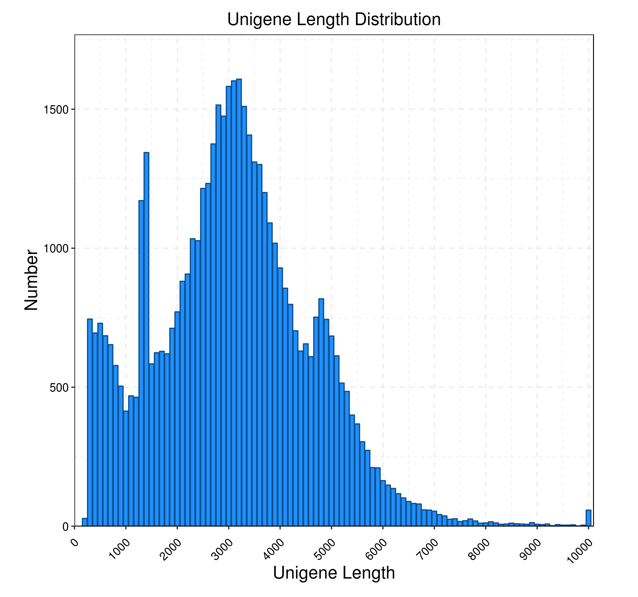


## Figure S6. Length distribution of unigenes in transcriptome.


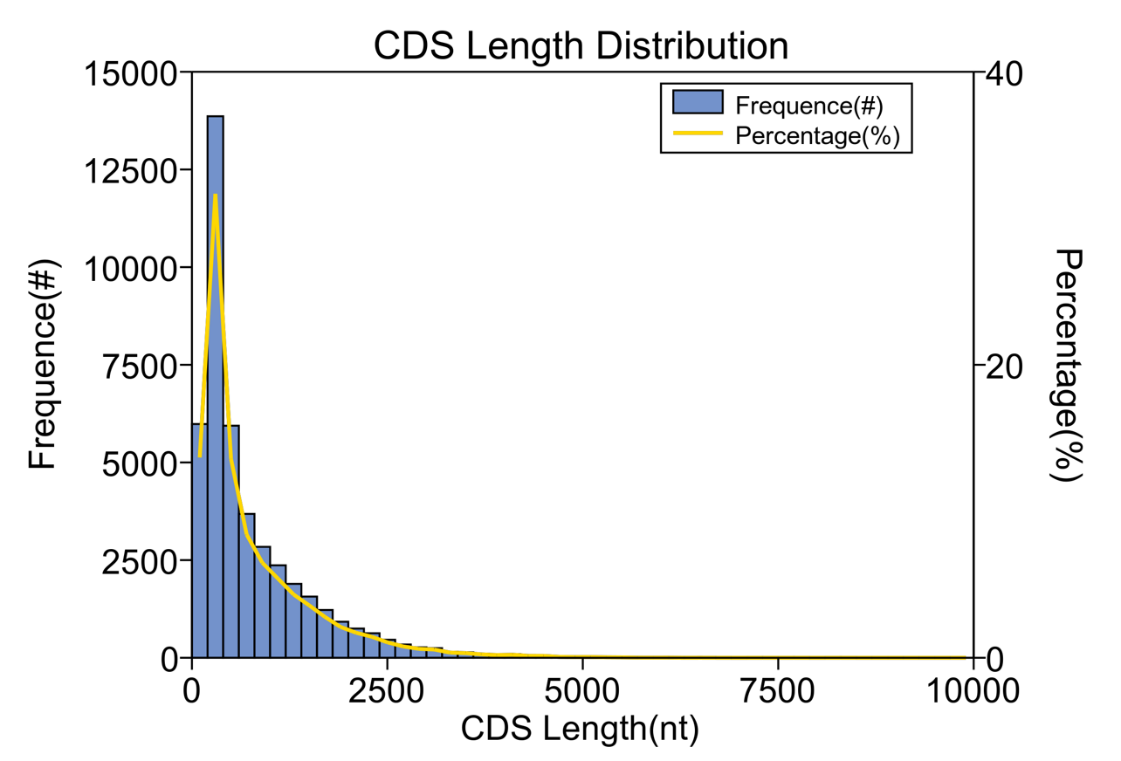


## Figure S7. Length distribution of coding DNA sequence (CDS) in transcriptome.


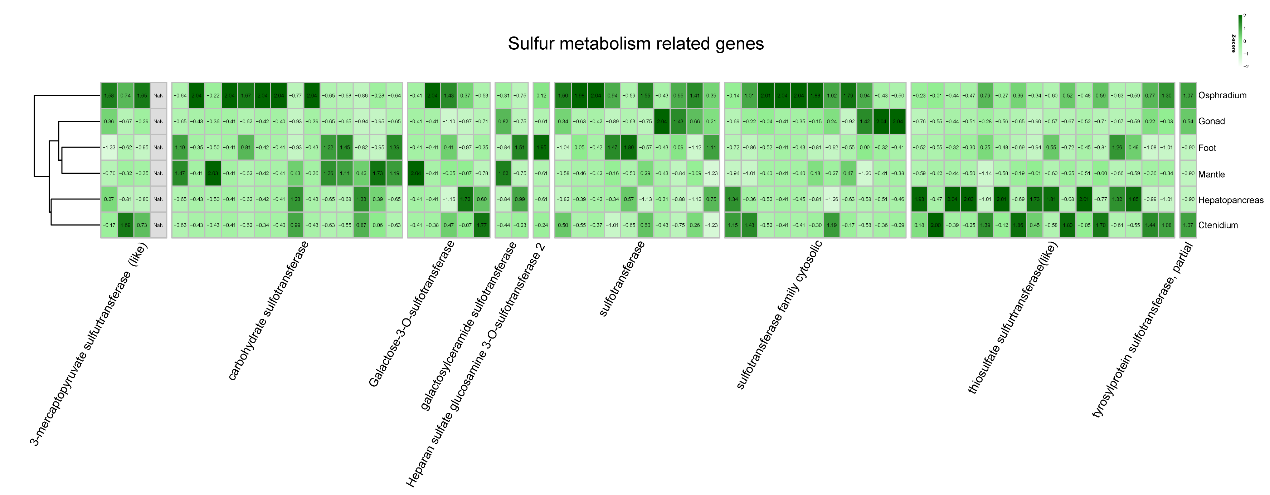


## Figure S8. Expression of sulfur metabolism related genes in different tissues.

(a) Expression of sulfurtransferase in different tissues.


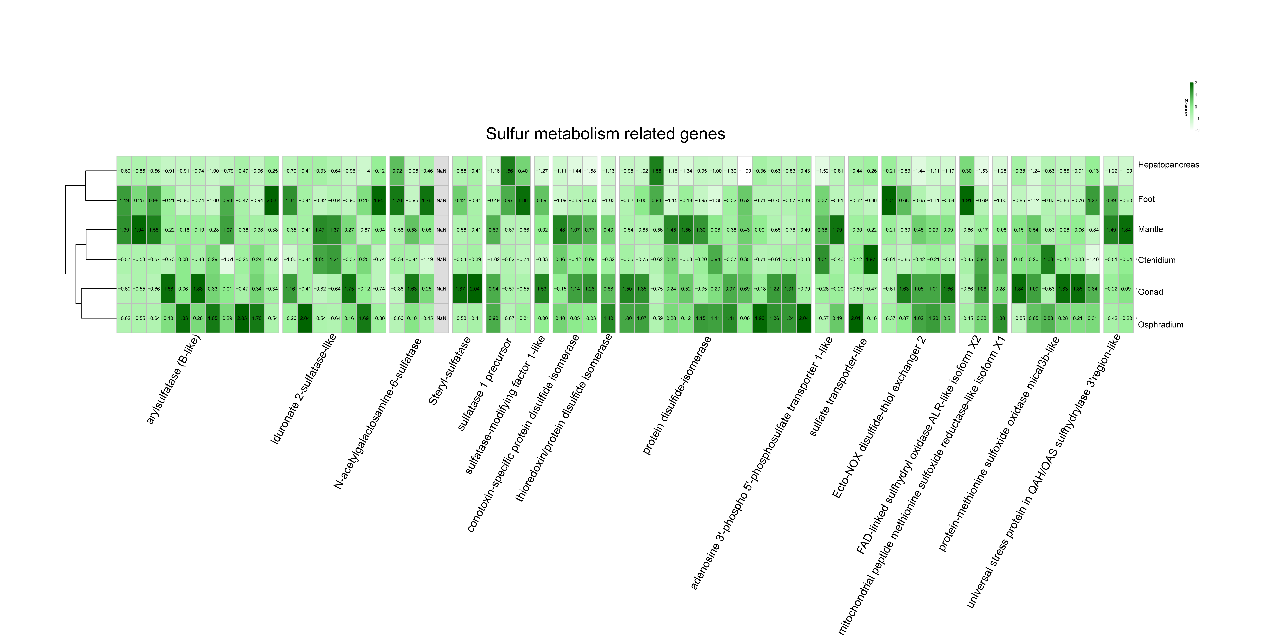


(b) Expression of other sulfur metabolism related genes in different tissues.

## Figure S9. Module eigengene *E* of gene co-expression networks.

1. Module eigengene *E* in hepatopancreas.

1. Module eigengene *E* in Ctenidium.


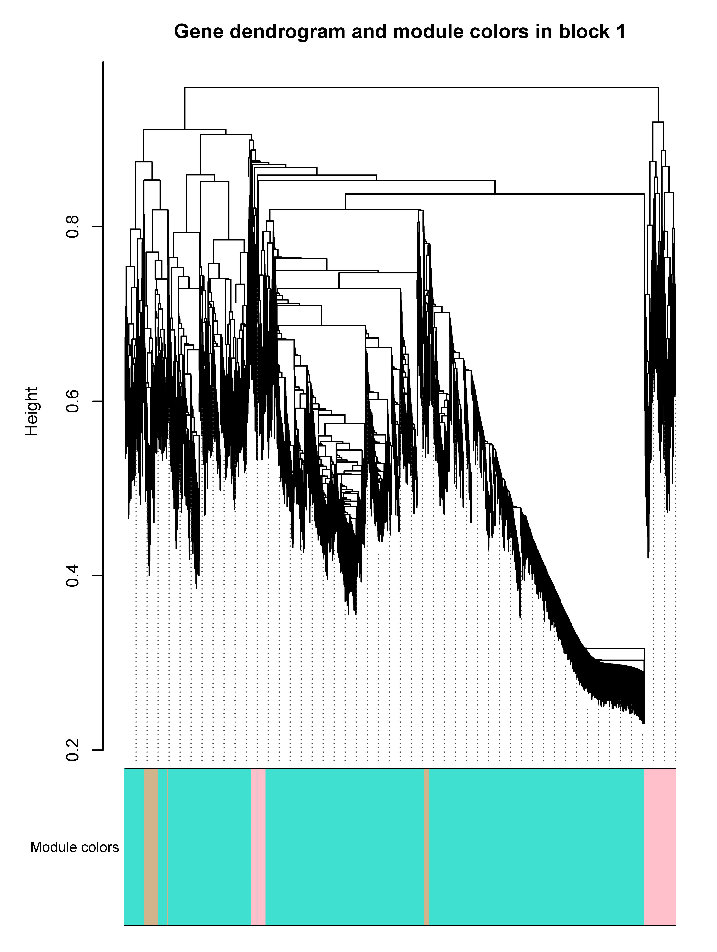


## Figure S10. Gene dendrograms and module colors of gene co-expression networks.

1. Gene dendrograms and module colors in block1.


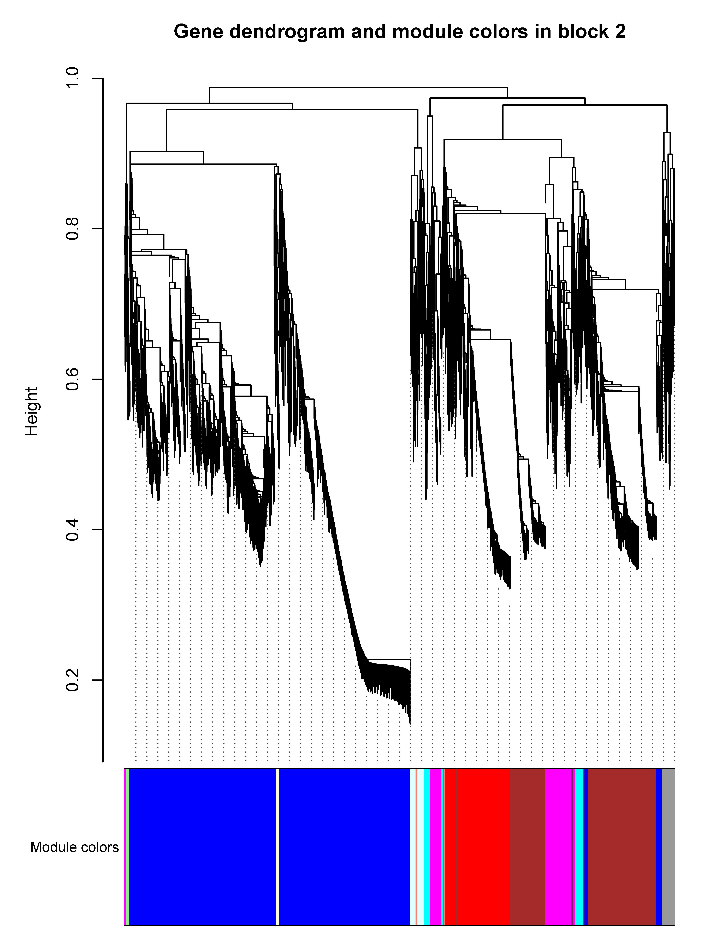


1. Gene dendrograms and module colors in block2.


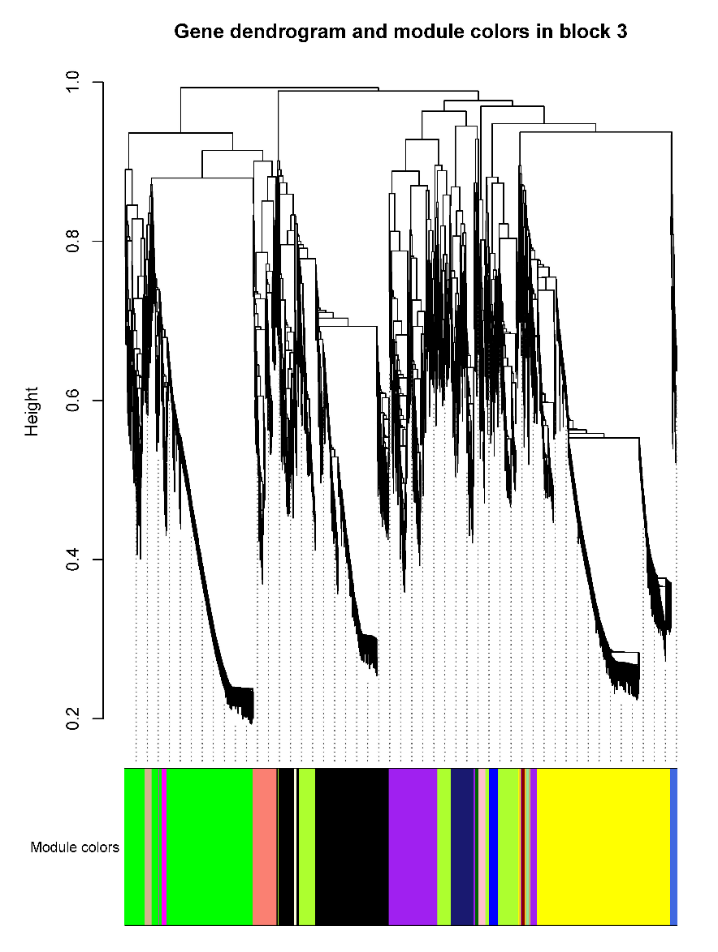


1. Gene dendrograms and module colors in block3.


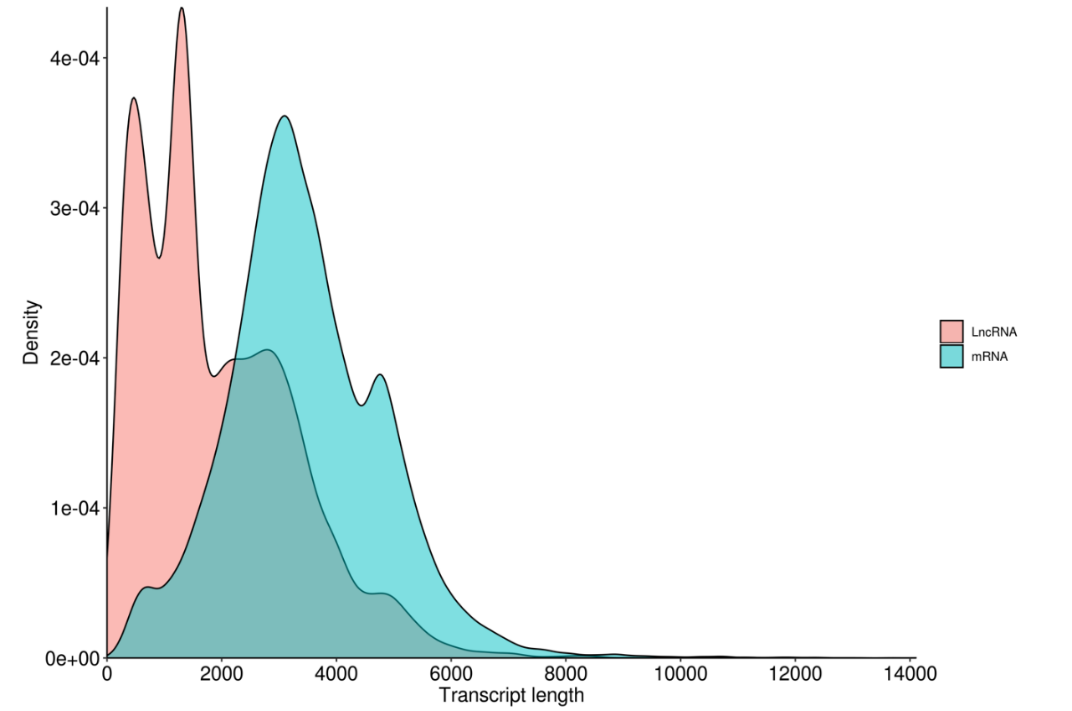


Figure S11. The distribution of transcript length of lncRNAs and mRNAs.


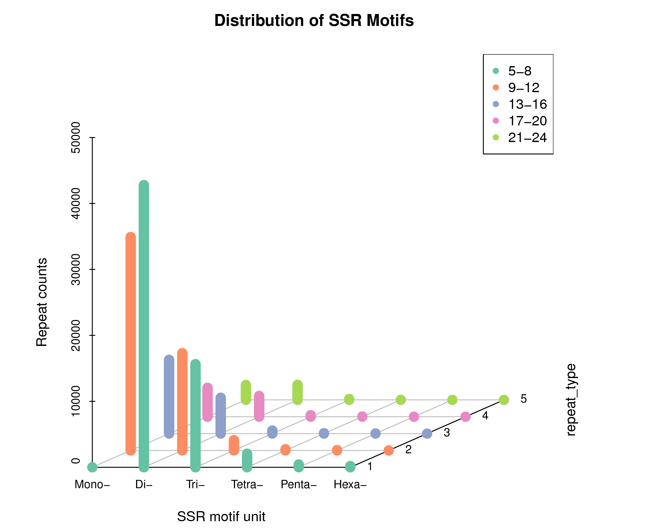


## Figure S12. The distribution of SSR motifs in transcriptome.


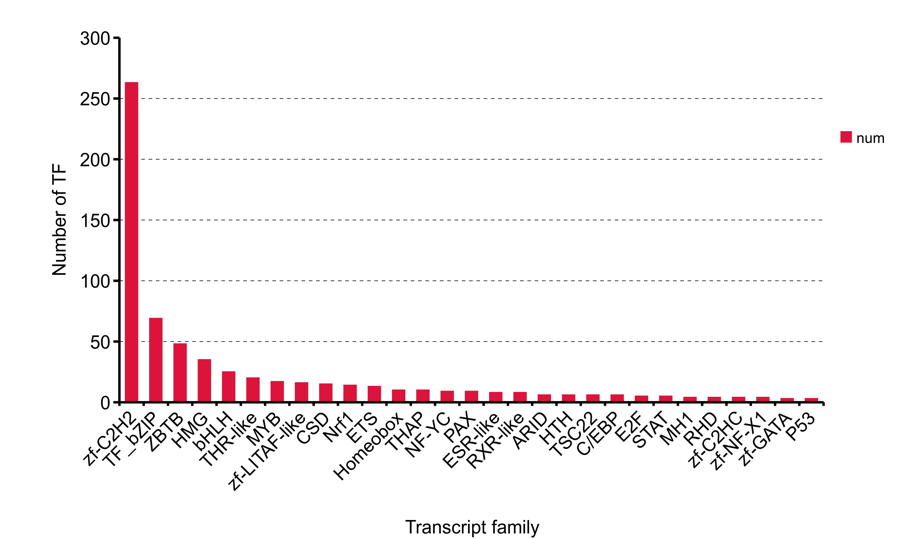


## **Figure S13. The numbers of transcription factors involved in the top transcription factor families of transcriptome.**

The x-axis represents the top transcription factor families and the y-axis represents the numbers of transcription factors.

# Supplementary Tables

## Table S1. Illumina statistics of the genome sequencing data of *P. buccinoides*.

|  |  |  |  |  |  |  |  |
| --- | --- | --- | --- | --- | --- | --- | --- |
| **Pair-end Lib** | **Insert Size (bp)** | **Raw Base (Gb)** | **Sequencing Length (bp)** | **Q20（%）** | **Q30（%）** | **GC (%)** | **Clean Base (Gb)** |
| NovaSeq 6000 | 350 | 26.1 | 150 | 94.6 | 89.7 | 42.4% | 21.0 |
| NovaSeq 6000 | 350 | 26.1 | 150 | 90.7 | 82.3 | 44.0% | 21.0 |
| **Total** |  | **52.2** |  |  |  |  | **42.0** |
|  |  |  |  |  |  |  |  |

## Table S2. PacBio statistics of the genome sequencing data of *P. buccinoides*.

|  |  |  |  |  |  |  |  |  |  |
| --- | --- | --- | --- | --- | --- | --- | --- | --- | --- |
| **Insert Library ID** | **Total Polymerase Bases (bp)** | **Total Subreads Bases (bp)** | **Number of Subreads** | **Mean Subreads Length** | **Subreads N50 Length** | **Total Fastq Reads Number** | **Total Fastq Base (bp)** | **Fastq Reads Length** | **GC Content (%)** |
| m54136_190323_092053 | 9,401,925,461 | 9,385,370,338 | 893,309 | 10,506 | 14,473 | 893,309 | 9,385,370,338 | 90,058 | 42.9 |
| m54139_190328_100120 | 9,476,387,137 | 9,452,744,969 | 830,243 | 11,386 | 14,226 | 830,243 | 9,452,744,969 | 161,363 | 42.8 |
| m54139_190402_055949 | 9,120,358,251 | 9,098,453,390 | 815,706 | 11,154 | 14,218 | 815,706 | 9,098,453,390 | 103,074 | 42.7 |
| m54139_190420_162333 | 9,249,057,995 | 9,231,371,016 | 975,366 | 9,465 | 13,799 | 975,366 | 9,231,371,016 | 87,304 | 42.8 |
| m54061_190424_180006 | 9,829,882,642 | 9,811,823,821 | 988,440 | 9,927 | 14,276 | 988,440 | 9,811,823,821 | 79,847 | 42.9 |
| m54061_190425_095352 | 9,654,073,290 | 9,636,424,465 | 966,234 | 9,973 | 14,286 | 966,234 | 9,636,424,465 | 86,021 | 42.8 |
| m54061_190425_200713 | 9,461,597,345 | 9,443,180,465 | 987,405 | 9,564 | 13,952 | 987,405 | 9,443,180,465 | 79,439 | 42.8 |
| m54061_190428_192830 | 9,392,683,856 | 9,374,248,331 | 972,108 | 9,643 | 14,053 | 972,108 | 9,374,248,331 | 76,587 | 42.8 |
| m54061_190430_195608 | 9,475,209,429 | 9,457,902,718 | 943,496 | 10,024 | 14,184 | 943,496 | 9,457,902,718 | 68,849 | 42.9 |
| m54061_190501_060906 | 9,005,014,787 | 8,989,157,824 | 879,005 | 10,227 | 14,349 | 879,005 | 8,989,157,824 | 71,387 | 42.8 |
| m54061_190501_162224 | 9,142,310,892 | 9,125,124,275 | 922,287 | 9,894 | 14,231 | 922,287 | 9,125,124,275 | 84,344 | 42.9 |
| m54061_190502_023540 | 9,431,276,181 | 9,411,871,668 | 1,001,489 | 9,398 | 13,755 | 1,001,489 | 9,411,871,668 | 83,242 | 42.8 |
| m54061_190502_125317 | 8,604,293,075 | 8,587,750,582 | 903,175 | 9,508 | 13,925 | 903,175 | 8,587,750,582 | 82,247 | 42.9 |
| m54061_190502_231115 | 9,149,958,032 | 9,131,240,034 | 943,136 | 9,682 | 13,974 | 943,136 | 9,131,240,034 | 77,203 | 42.8 |
| **Total/Mean** | **130,394,028,373** | 130,136,663,896 | 13,021,399 | **10,025** | 14,122 |  | **130,136,663,896** | 87,926 |  |
|  |  |  |  |  |  |  |  |  |  |

## Table S3. Hi-C statistics of the genome sequencing data of *P. buccinoides*.

|  |  |  |  |  |  |  |
| --- | --- | --- | --- | --- | --- | --- |
| **Sample** | **Raw Read Number** | **Raw Base(G)** | **Q30(%)** | **GC Content (%)** | **Sequence coverage (X)** | **Clean Base (Gb)** |
| LG-1 | 58,575,920 | 8.8 | 90.4;91.4 | 42.9;43.3 | 4 | 7.4 |
| LG-2 | 45,816,540 | 6.9 | 90.8;89.3 | 42.8;43.1 | 3 | 5.9 |
| LG-1 | 1,520,032,908 | 228.0 | 90.5;86.6 | 42.8;43.6 | 109 | 188.1 |
| LG-2 | 994,552,884 | 149.2 | 90.9;88.2 | 42.7;43.3 | 71 | 125.7 |
| **Total** | 2,618,978,252 | **392.9** |  |  | **187** | **327.1** |
|  |  |  |  |  |  |  |

## Table S4a. Statistics of *P. buccinoides* Illumina transcriptome reads (Raw data).

|  |  |  |  |  |  |  |
| --- | --- | --- | --- | --- | --- | --- |
| **Library ID** | **Sample name** | **Raw Read Number** | **Raw Base (bp)** | **Q20(%)** | **Q30(%)** | **GC content (%)** |
| BRAS190322624-1A | Hepatopancreas | 27,138,070 | 4,070,710,500 | 97.4 | 93.2 | 37.0 |
| BRAS190322636-1A | Hepatopancreas | 27,138,070 | 4,070,710,500 | 96.6 | 91.5 | 37.2 |
| BRAS190323385-1A | Foot | 23,822,652 | 3,573,397,800 | 96.9 | 92.1 | 39.5 |
| BRAS190322630-1A | Foot | 23,822,652 | 3,573,397,800 | 96.6 | 91.3 | 39.5 |
| BRAS190322622-1A | Mantle | 32,757,351 | 4,913,602,650 | 97.0 | 92.7 | 41.8 |
| BRAS190322632-1A | Mantle | 32,757,351 | 4,913,602,650 | 96.3 | 91.3 | 42.0 |
| BRAS190322620-1A | Ctenidium | 30,429,426 | 4,564,413,900 | 97.3 | 93.1 | 40.4 |
| BRAS190322638-1A | Ctenidium | 30,429,426 | 4,564,413,900 | 96.6 | 91.6 | 40.5 |
| BRAS190322639-1A | Gonad | 20,675,657 | 3,101,348,550 | 96.6 | 91.9 | 40.7 |
| BRAS190322634-1A | Gonad | 20,675,657 | 3,101,348,550 | 95.6 | 89.8 | 40.9 |
| BRAS190322628-1A | Osphradium | 23,534,699 | 3,530,204,850 | 96.4 | 91.5 | 40.0 |
| BRAS190323384-1A | Osphradium | 23,534,699 | 3,530,204,850 | 94.6 | 88.0 | 40.2 |
|  |  |  |  |  |  |  |

## Table S4b. Statistics of *P. buccinoides* Illumina transcriptome reads (Clean data).

|  |  |  |  |  |  |  |
| --- | --- | --- | --- | --- | --- | --- |
| **Library ID** | **Sample name** | **Clean Read Number** | **Clean Base (bp)** | **Q20(%)** | **Q30(%)** | **GC content (%)** |
| BRAS190322624-1A | Hepatopancreas | 26,111,872 | 3,916,780,800 | 97.5 | 93.3 | 36.8 |
| BRAS190322636-1A | Hepatopancreas | 26,111,872 | 3,916,780,800 | 97.0 | 92.0 | 37.0 |
| BRAS190323385-1A | Foot | 22,356,076 | 3,353,411,400 | 97.1 | 92.3 | 39.3 |
| BRAS190322630-1A | Foot | 22,356,076 | 3,353,411,400 | 96.9 | 91.7 | 39.4 |
| BRAS190322622-1A | Mantle | 31,407,874 | 4,711,181,100 | 97.2 | 92.9 | 41.6 |
| BRAS190322632-1A | Mantle | 31,407,874 | 4,711,181,100 | 96.7 | 91.7 | 41.8 |
| BRAS190322620-1A | Ctenidium | 29,747,376 | 4,462,106,400 | 97.5 | 93.3 | 40.3 |
| BRAS190322638-1A | Ctenidium | 29,747,376 | 4,462,106,400 | 96.9 | 92.0 | 40.4 |
| BRAS190322639-1A | Gonad | 20,056,417 | 3,008,462,550 | 96.8 | 92.1 | 40.6 |
| BRAS190322634-1A | Gonad | 20,056,417 | 3,008,462,550 | 96.0 | 90.2 | 40.8 |
| BRAS190322628-1A | Osphradium | 22,772,417 | 3,415,862,550 | 96.6 | 91.8 | 39.8 |
| BRAS190323384-1A | Osphradium | 22,772,417 | 3,415,862,550 | 95.1 | 88.5 | 40.1 |
|  |  |  |  |  |  |  |

## Table S5. Statistics of *P. buccinoides* Iso transcriptome reads.

|  |  |
| --- | --- |
|  | **Size/Number/Other** |
| **Library ID** | ISO0785 |
| **Name** | m54219_190116_081412.subreads.bam.fq.gz |
| **Polymerase Read Number** | 387,866 |
| **Polymerase Read Base (Gb)** | 21.3 |
| **Average Polymerase Read Length (bp)** | 54,805 |
| **Polymerase Read N50 (bp)** | 89,182 |
| **Subreads Read Number** | 11,712,719 |
| **Subreads Base (Gb)** | 20.4 |
| **Average Subreads Length (bp)** | 1,740 |
| **Subreads N50 (bp)** | 2,526 |
| **Subreads GC content (%)** | 37.73 |
| **CCS number** | 328,648 |
| **CCS N50 (bp)** | 3,138 |
| **Average CCS Length (bp)** | 2,492 |
| **Max CCS length (bp)** | 14,396 |
| **Min CCS length (bp)** | 50 |
|  |  |

## Table S6. Transcriptome sequencing data of *P. buccinoides* (for aiding gene annotation).

|  |  |  |  |  |  |  |
| --- | --- | --- | --- | --- | --- | --- |
| **Pair-end Lib** | **Raw Read Number** | **Raw Base (Gb)** | **Q30 (%)** | **GC Content (%)** | **Reads Length (bp)** | **Clean Base (Gb)** |
| NovaSeq6000 | 22,431,750 | 3.4 | 92.7 | 39.5% | 150 | 3.0 |
| NovaSeq6000 | 22,431,750 | 3.4 | 90.7 | 39.6% | 150 | 3.0 |
| **Total** | **44,863,500** | **6.8** |  |  |  | **6.0** |
|  |  |  |  |  |  |  |

## Table S7. Summary statistics of the genome sequencing data of *P. buccinoides*.

|  |  |  |  |  |
| --- | --- | --- | --- | --- |
| **Pair-end Lib** | **Insert Size (bp)** | **Raw Base (Gb)** | **Reads Length (bp)** | **Sequence Coverage(X)** |
| NovaSeq 6000 reads | 350 | 52.2 | 150 | 24.9 |
| PacBio reads | 20,000 | 130.4 | 14,000 | 62.1 |
| Hi-C | 300-500 | 392.9 | 150 | 187.0 |
| **Total** |  | **575.5** |  | **274.0** |
|  |  |  |  |  |

## Table S8. Contig assembly of the *P. buccinoides* genome using Illumina and PacBio reads. Related to Figure 1e.

|  |  |  |
| --- | --- | --- |
|  | **Contig** | |
|  | **Size(bp)** | **Number** |
| N90 | 38,389 | 10,194 |
| N80 | 68,170 | 5,999 |
| N70 | 128,507 | 3,714 |
| N60 | 217,069 | 2,461 |
| N50 | 308,656 | 1,643 |
| Longest | 3,988,471 | |
| Total Size | 2,114,588,964 | |
| Total number (>=100bp) | 18,749 | |
| Total number (>=2kb) | 18,735 | |
| N% | 0% | |
| GC rate | 42.8% | |
| Busco | 86.0% | |
|  |  |  |

## Table S9. Summary statistics of the *P. buccinoides* chromosomal-level genome assembly. Related to Figure 1d, e.

|  |  |  |
| --- | --- | --- |
|  | **Size (bp)/Number** | |
| **Number of chromosomes** | 34 | |
| **Scaffold N50 of genome size (bp)/Number** | 55,611,335 | 16 |
| **Scaffold N90 of genome size (bp)/Number** | 34,010,429 | 34 |
| **Total number (>=2kb) of genome** | 4,237 | |
| **Scaffold N50 of pseudochromosomes size (bp)/Number** | 59,964,442 | 14 |
| **Scaffold N90 of pseudochromosomes size (bp)/Number** | 41,595,758 | 29 |
| **Maximum scaffold size (bp)** | 105,778,436 | |
| **Total size (bp) of pseudochromosomes** | 1,938,116,449 | |
| **Total size (bp) of genome** | 2,123,789,595 | |
| **N%** | 0.4% | |
|  |  |  |

## Table S10. Prediction of repeat elements in the *P. buccinoides* genome. Related to Figure 1c, S1.

|  |  |  |
| --- | --- | --- |
| **Type** | **Repeat Size (bp)** | **% of genome** |
| RepeatMask | 448,316,916 | 21.2 |
| ProteinMask | 110,520,182 | 5.2 |
| TRF | 556,276,320 | 26.3 |
| De novo | 1,086,211,243 | 51.4 |
| **Total** | 1,552,924,576 | **73.4** |
|  |  |  |

Note: The interspersed repeats and tandem repeats were predicted in the *P. buccinoides* genome. The interspersed repeats were predicted by RepeatMasker and ProteinMask. The tandem repeats were predicted by TRF (Tandem repeats finder).

## Table S11. Categories of repeat elements predicted in the *P. buccinoides* genome. Related to Figure 1c, S1.

|  |  |  |  |  |  |  |
| --- | --- | --- | --- | --- | --- | --- |
|  | **Repbase TEs** | | **TE protiens** | | **Combined TEs** | |
| **Type** | **Length (bp)** | **% in genome** | **Length (bp)** | **% in genome** | **Length (bp)** | **% in genome** |
| DNA | 389,100,363 | 18.40 | 7,906,994 | 0.37 | 511,613,873 | 24.19 |
| LINE | 109,969,244 | 5.20 | 92,936,183 | 4.39 | 337,698,234 | 15.97 |
| SINE | 571,066 | 0.03 | 0 | 0.00 | 36,818,909 | 1.74 |
| LTR | 103,126,934 | 4.88 | 9,693,424 | 0.46 | 149,297,997 | 7.06 |
| Other | 66,162 | 0.00 | 0 | 0.00 | 66,162 | 0.00 |
| Unknown | 0 | 0.00 | 0 | 0.00 | 530,131,647 | 25.07 |
| **Total** | **448,316,916** | **21.20** | **110,520,182** | **5.23** | **1,266,758,454** | **59.91** |
|  |  |  |  |  |  |  |

Note: “Other” refer to the repeats that can be classified by RepeatMasker, but not included by the classes above; “Unknown” refer to the repeats that can’t be classified by RepeatMasker.

## Table S12. Prediction of gene structure in *P. buccinoides* genomes. Related to Figure 1e.

|  |  |  |  |  |  |  |  |
| --- | --- | --- | --- | --- | --- | --- | --- |
|  | **Gene Set** | **Number** | **Average Transcript Length (bp)** | **Average CDS Length (bp)** | **Average Exons per Gene** | **Average Exon Length (bp)** | **Average Intron Length (bp)** |
| ***De novo*** | Augustus | 72,249 | 10,650 | 1,027 | 4 | 233 | 2,820 |
|  | Genscan | 118,974 | 10,858 | 1,178 | 5 | 260 | 2,747 |
|  | Snap | 132,616 | 8,727 | 722 | 4 | 182 | 2,691 |
| **Homolog** | *Aplysia californica* | 52,131 | 4,489 | 800 | 2 | 325 | 2,526 |
|  | *Biomphalaria glabrata* | 86,575 | 2,707 | 656 | 2 | 353 | 2,393 |
|  | *Caenorhabditis elegans* | 230 | 5,083 | 639 | 3 | 241 | 2,683 |
|  | *Crassostrea gigas* | 42,154 | 4,973 | 948 | 3 | 362 | 2,491 |
|  | *Crassostrea virginica* | 6,517 | 3,470 | 1,005 | 2 | 468 | 2,148 |
|  | *Drosophila melanogaster* | 3,545 | 6,658 | 850 | 3 | 303 | 3,222 |
|  | *Mizuhopecten yessoensis* | 8,740 | 2,589 | 794 | 2 | 427 | 2,088 |
|  | *Lottia gigantea* | 119,767 | 2,108 | 571 | 2 | 339 | 2,242 |
|  | *Octopus bimaculoides* | 39,211 | 4,080 | 677 | 2 | 307 | 2,827 |
|  | *Pomacea canaliculata* | 17,381 | 9,335 | 1,099 | 4 | 270 | 2,680 |
| **Transcriptome** | Cufflinks | 19,223 | 12,649 | 2,233 | 6 | 379 | 2,130 |
| **MAKER** |  | 45,545 | 11,043 | 1,346 | 5 | 274 | 2,477 |
|  |  |  |  |  |  |  |  |

## Table S13. Functional annotation of the predicted protein-coding genes in the *P. buccinoides*. Related to Figure 2a.

|  | **Annotated gene number** | **% of gene** |  |  |  |
| --- | --- | --- | --- | --- | --- |
| Total | 45,545 | 100.0 |  |  |  |
| Nr-Annotated | 41,180 | 90.4 |  |  |  |
| Swissprot-Annotated | 27,244 | 59.8 |  |  |  |
| KEGG-Annotated | 34,527 | 75.8 |  |  |  |
| GO-Annotated | 12,447 | 27.3 |  |  |  |
| COG-Annotated | 9,524 | 20.9 |  |  |  |
| TrEMBL-Annotated | 41,363 | 90.8 |  |  |  |
| Interpro-Annotated | 27,367 | 60.1 |  |  |  |
| Overall | 42,162 | 92.6 |  |  |  |
|  | | | | |  |

## Table S14. Statistics of gene families in 11 examined species.

| **Species** | **Genes in** | **Unclustered genes** | **Family** | **Unique** | **Average** |
| --- | --- | --- | --- | --- | --- |
|  | **families** |  | **number** | **families** | **genes per** |
|  |  |  |  |  | **family** |
| *Aplysia californica* | 22,839 | 4,497 | 12,042 | 743 | 1.9 |
| *Biomphalaria glabrata* | 30,883 | 5,779 | 13,648 | 1,507 | 2.3 |
| *Crassostrea gigas* | 42,805 | 3,797 | 14,398 | 1,949 | 3.0 |
| *Drosophila melanogaster* | 28,681 | 2,036 | 8,730 | 2,425 | 3.3 |
| *Helobdella robusta* | 15,751 | 7,675 | 8,166 | 671 | 1.9 |
| *Lingula anatina* | 38,822 | 2,557 | 12,477 | 1,893 | 3.1 |
| *Lottia gigantea* | 19,554 | 4,277 | 12,770 | 641 | 1.5 |
| *Mizuhopecten yessoensis* | 38,470 | 3,086 | 14,492 | 1,409 | 2.7 |
| *Octopus bimaculoides* | 21,791 | 2,203 | 10,284 | 725 | 2.1 |
| *Phymorhynchus buccinoides* | 39,151 | 6,394 | 12,754 | 1,475 | 3.1 |
| *Pomacea canaliculata* | 38,831 | 1,547 | 13,215 | 1,226 | 2.9 |
